# Dibohemamines I–O from *Streptomyces* sp. GZWMJZ-662, an endophytic actinomycete from the medicinal and edible plant *Houttuynia cordata* Thunb.

**DOI:** 10.1007/s13659-024-00494-4

**Published:** 2025-01-06

**Authors:** Dong-Yang Wang, Ming-Xing Li, Yan-Chao Xu, Peng Fu, Wei-Ming Zhu, Li-Ping Wang

**Affiliations:** 1 State Key Laboratory of Functions and Applications of Medicinal Plants, Guizhou Medical University, Guiyang, 550014 China; 2 Natural Product Research Center of Guizhou Province, Guiyang, 550014 China; 3 School of Pharmaceutical Sciences, Guizhou Medical University, Guiyang, 561113 China; 4 School of Medicine and Pharmacy, Ocean University of China, Qingdao, 266003 China

**Keywords:** Endophytic actinomycete, Secondary metabolite, Dibohemamine, Cytotoxicity

## Abstract

A chemical investigation of *Streptomyces* sp. GZWMJZ-662, an endophytic actinomycete isolated from *Houttuynia cordata* Thunb., has yielded eleven bohemamine dimers (**1**–**11**). Notably, the newly identified dibohemamines I–O (**1**–**7**) have not been previously reported. Their structures were elucidated through detailed spectroscopic analysis, encompassing high-resolution electrospray ionization mass, nuclear magnetic resonance, infrared radiation, ultraviolet–visible, and electronic circular dichroism spectroscopy. Dibohemamine I (**1**) exhibited selective cytotoxic effects against the cancer cell lines 786-O and GBC-SD among the 18 cell lines evaluated, with the half-inhibitory concentration values of 3.24 ± 0.20 and 7.36 ± 0.41 μM, respectively.

**Graphical Abstract:**

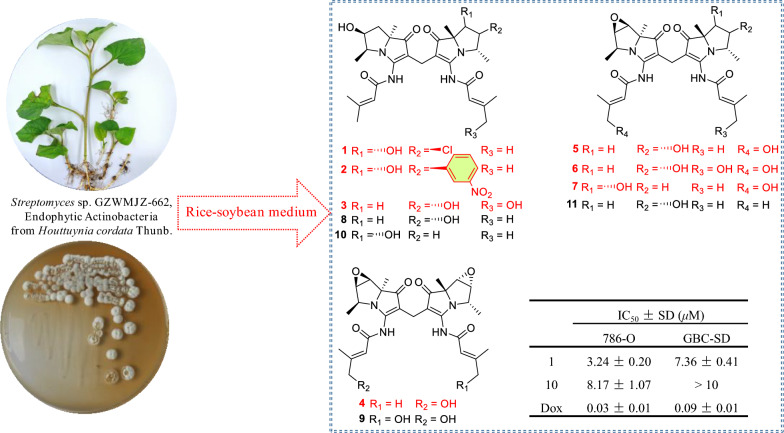

**Supplementary Information:**

The online version contains supplementary material available at 10.1007/s13659-024-00494-4.

## Introduction

Actinomycetes, a group of bacteria thriving in the diverse environments of natural ecosystems, produced many biologically active metabolites [1–3]. The bacterial density of microbial communities that inhabit the soil and rhizosphere around plant roots, can reach up to 10^6^ cells per cubic millimeter, and actinomycetes account for a notable 4% of the total population [4]. The capacity of endophytic actinomycetes to synthesize compounds with innovative structures and remarkable bioactivity has been well documented, which is a testament to their potential research area in the field of natural product chemistry [5, 6]. Despite their proven capabilities, endophytic actinomycetes remain a relatively unexplored group within the microbial world, with much of their potential still shrouded in mystery [7]. Intriguingly, studies suggest that these endophytic bacteria are capable of producing metabolites with pharmacological activities that closely resemble, or even exactly match those of their host plants [8]. *Houttuynia cordata* Thunb. is a food and medicine homology plant with diverse phytochemical constituents, which contribute to its wide range of biological activities [9]. The antitumor effects of its extract have been extensively studied, highlighting its potential as a source of therapeutic agents [10–13]. However, metabolites produced by endophytic actinomycetes within *H. cordata* have not yet been fully studied.

**Fig. 1:**
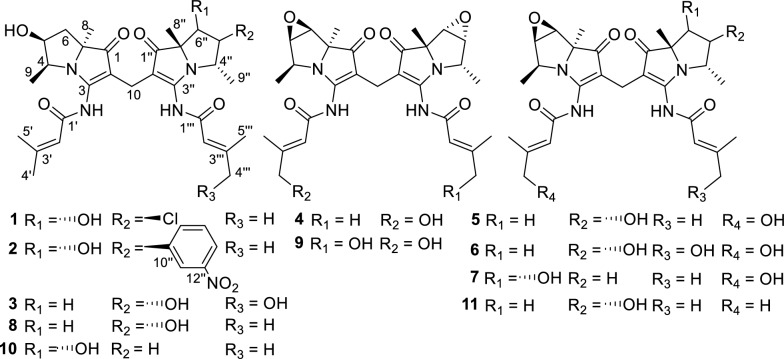
The chemical structures of compounds **1** − **11**

During our research, furanpydones A and B with potent cytotoxic activity have been identified from an endophytic fungus in *H. cordata* [14]. Our ongoing study on antitumor compounds focuses on the metabolites of endophytic actinomycetes within *H. cordata*. Further exploration of *Streptomyces* sp. GZWMJZ-662 yielded seven novel bohemamine dimers, dibohemamines I–O (**1–7**) (Fig. 1). Four known dibohemamines were also identified, including dibohemamine B (**8**) [15], dibohemamine G (**9**) [16], dibohemamine C (**10**) [15], and dibohemamine F (**11**) [17].

## Results and discussions

**Fig. 2:**
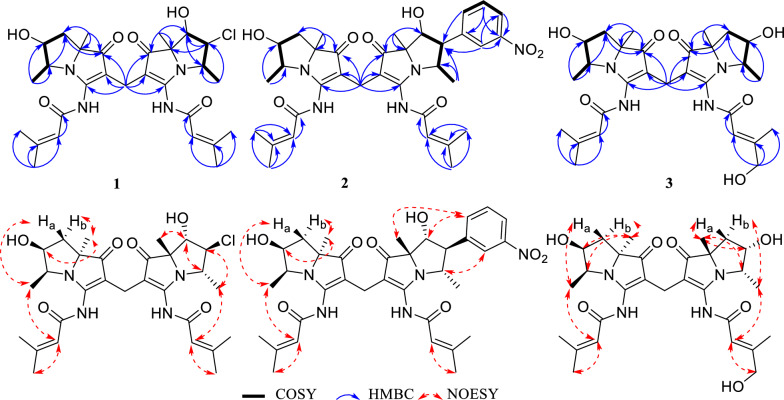
Key 2D NMR correlations of dibohemamines I–K (**1**–**3**)

The molecular formula of **1** was determined as C_29_H_39_O_6_N_4_Cl via HR-ESIMS (Fig. S2). The IR spectrum (Fig. S1) indicate that **1** contains methylene group (2930, 1451 cm^–1^), *α*,*β*-unsaturated ketone (1692 cm^–1^), and amide group (1646, 1554 cm^–1^) [15]. The ultraviolet–visible (UV–Vis) spectrum of **1** showed peaks at 248, 285, and 346 nm, similar to those of bohemamines and dibohemamines [15, 18]. The comparison of the NMR data of **1** (Table 1, Figs. S3 and S4) with that of bohemamines and dibohemamines indicates that **1** should be an analog of dibohemamine containing one bohemamine B moiety and one 5-Cl-bohemamine C moiety [18]. The bohemamine B moiety in **1** was confirmed via the spin system of H_2_-6/H-5/H-4/H_3_-9, and the key HMBC from H_3_-8 to C-1/C-6/C-7, from H_3_-9 to C-4/C-5, and from H-2ʹ to C-1ʹ/C-3ʹ/C-4ʹ/C-5ʹ, as well as the NOESY correlation of H-2ʹ to H_3_-9 (Fig. 2). The 5-Cl-bohemamine C moiety of **1** was confirmed via the spin system of H-6ʹʹ/H-5ʹʹ/H-4ʹʹ/H_3_-9ʹʹ, HMBC from H_3_-8ʹʹ to C-1ʹʹ/C-6ʹʹ/C-7ʹʹ, from H_3_-9ʹʹ to C-4ʹʹ/C-5ʹʹ, and from H-2ʹʹʹ to C-1ʹʹʹ/C-3ʹʹʹ/C-4ʹʹʹ/C-5ʹʹʹ, and the key NOESY correlation of H-2ʹʹʹ to H_3_-9ʹʹ. These two bohemamines connected through a methylene carbon between C-2 and C-2″, which was confirmed by the HMBC from H_2_-10 to C-1/C-2/C-3/C-1″/C-2″/C-3″ [17]. Thus, the skeleton of compound **1** was confirmed and was designated as dibohemamine I.

**Table 1:** The ^1^H (600 MHz) and ^13^C (150 MHz) NMR Data of **1**–**3** in CD_3_OD

No.	**1**^a^		**2**^a^		**3**^a^	
	*δ*_C_, type	*δ*_H_, mult. (*J* in Hz)	*δ*_C_, type	*δ*_H_, mult. (*J* in Hz)	*δ*_C_, type	*δ*_H_, mult. (*J* in Hz)
1	204.8, C		204.3, C		204.4, C	
2	106.3, C		109.4, C		104.9, C	
3	166.9, C		166.7, C		166.7, C	
4	60.2, CH	4.04, dq (6.9, 6.7)	60.3, CH	4.06, qd (6.7, 6.0)	60.3, CH	4.06, qd (6.7, 5.9)
5	73.9, CH	4.72, ddd (11.6, 6.9, 5.7)	70.1, CH	4.73, ddd (11.4, 6.0, 5.9)	74.2, CH	4.71, ddd (11.3, 5.9, 5.7)
6	36.7, CH_2_	1.56, t (11.6) 1.99, dd (11.6, 5.7)	37.0, CH_2_	1.61, t (11.4) 2.00, dd (11.4, 5.9)	37.0, CH_2_	1.60, t (11.3) 2.00, dd (11.3, 5.7)
7	74.2, C		74.2, C		74.2, C	
8	25.9, CH_3_	1.38, s	25.9, CH_3_	1.39, s	25.8, CH_3_	1.38, s
9	10.3, CH_3_	0.81, d (6.7)	10.4, CH_3_	0.89, d (6.7)	10.3, CH_3_	0.88, d (6.7)
10	13.4, CH_2_	2.87, d (15.7) 2.82, d (15.7)	13.8, CH_2_	2.96, d (15.6) 2.91, d (15.6)	13.7, CH_2_	2.89, d (10.0) 2.87, d (10.0)
1ʹ	165.0, C		164.9, C		164.9, C	
2ʹ	118.3, CH	6.14 − 6.15, m	118.3, CH	6.13 − 6.14, m	118.1, CH	6.11 − 6.12, m
3ʹ	160.9, C		160.9, C		161.1, C	
4ʹ	27.8, CH_3_	2.04, d (1.1)	27.8, CH_3_	2.02, d (1.1)	27.8, CH_3_	2.03, d (0.9)
5ʹ	20.6, CH_3_	2.27, d (1.1)	20.6, CH_3_	2.26, d (1.1)	20.6, CH_3_	2.26, d (0.9)
1ʹʹ	200.9, C		203.2, C		204.6, C	
2ʹʹ	105.2, C		104.3, C		105.1, C	
3ʹʹ	166.4, C		166.6, C		166.7, C	
4ʹʹ	67.1, CH	4.23, q (6.9)	60.6, CH	4.77, qd (6.7, 2.5)	60.2, CH	4.06, qd (6.7, 5.9)
5ʹʹ	72.5, CH	4.21, br s	86.3, CH	5.21, dd (2.5, 2.4)	74.3, CH	4.71, ddd (11.3, 5.9, 5.7)
6ʹʹ	80.2, CH	4.20, br s	80.6, CH	4.46, d (2.4)	37.1, CH_2_	1.62, t (11.3) 2.00, dd (11.3, 5.7)
7ʹʹ	83.2, C		80.1, C		74.3, C	
8ʹʹ	23.9, CH_3_	1.66, s	26.0, CH_3_	1.19, s	25.9, CH_3_	1.39, s
9ʹʹ	19.1, CH_3_	1.26, d (6.9)	16.1, CH_3_	1.43, d (6.7)	10.3, CH_3_	0.89, d (6.7)
10ʹʹ			133.5, C			
11ʹʹ			147.1, CH	9.62, s		
12ʹʹ			146.8, C			
13ʹʹ			148.1, CH	9.14, d (8.0)		
14ʹʹ			130.0, CH	8.35, dd (8.0, 6.3)		
15ʹʹ			147.0, CH	9.32, d (6.3)		
1ʹʹʹ	164.8, C		165.3, C		165.1, C	
2ʹʹʹ	118.0, CH	6.11 − 6.12, m	118.3, CH	6.15 − 6.16, m	115.3, CH	6.39 − 6.40, m
3ʹʹʹ	161.3, C		161.4, C		162.7, C	
4ʹʹʹ	27.7, CH_3_	2.04, d (1.1)	27.7, CH_3_	2.04, d (1.1)	67.4, CH_2_	4.14, br s
5ʹʹʹ	20.7, CH_3_	2.26, d (1.1)	20.7, CH_3_	2.29, d (1.1)	16.1, CH_3_	2.16, br s

^a^The chemical shifts of carbons were obtained via DEPTQ experiments

**Fig. 3:**
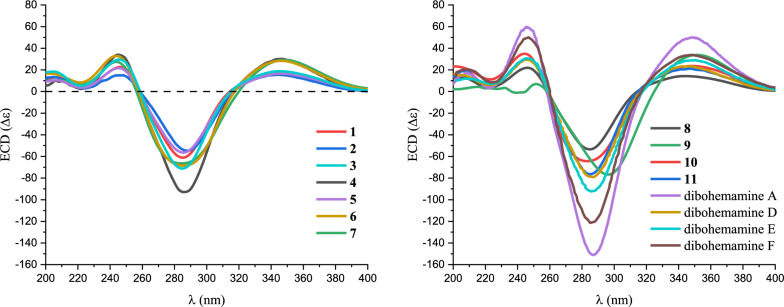
Experimental ECD spectra of compounds **1**–**11** and reported ECD spectra of dibohemamines A [15] and D–F [17]

The NOESY correlations (Fig. 2) of H_3_-9 to H-6a, H_3_-8 to H-5/H-6b, H-5′′ to H_3_-9′′, and H-6′′ to H-4′′/H_3_-8′′ suggest that the relative configurations of the two moieties are respectively identical to that of bohemamine B and 5-Cl-bohemamine C. Additionally, the absolute configuration of dibohemamine I was ascertained by comparing its ECD spectrum with those of reported dibohemamines (Fig. 3) [15–17].

The molecular formula of **2** was determined as C_35_H_43_O_8_N_5_ via the HR-ESIMS peak at *m*/*z* 662.3174 [M + H]^+^ (calcd for C_35_H_44_O_8_N_5_, 662.31844). The analysis of **2** via NMR data (Table 1; Fig. 2) reveals it as an analog of dibohemamine with one bohemamine B unit and one 5ʹʹ-substituted bohemamine C unit. These two bohemamines are connected through a methylene carbon between C-2 and C-2″ indicated by the HMBC from H_2_-10 to C-1/C-2/C-3/C-1″/C-2″/C-3″ [17]. Furthermore, the COSY correlations of H-13ʹʹ/H-14ʹʹ/H-15ʹʹ and the HMBC from H-11ʹʹ to C-12ʹʹ/C-13ʹʹ/C-15ʹʹ, and from H-14ʹʹ to C-10ʹʹ/C-12ʹʹ indicate the presence of a *meta*-disubstituted benzene. The connection between C-5ʹʹ in bohemamine C and C-10ʹʹ in disubstituted benzene was built via the HMBC correlations from H-11ʹʹ/H-15ʹʹ to C-5ʹʹ. The remaining one nitrogen and two oxygen atoms, along with the characteristic strong absorption bands at 1567 and 1383 cm^−1^ in IR spectrum [19], indicates that the C-12ʹʹ position is substituted by a nitro group. Consequently, the planar structure of **2** has been elucidated and is named dibohemamine J.

The NOESY of H_3_-8 to H-6b/H-5, H-6a to H_3_-9, H-15ʹʹ to H_3_-8ʹʹ/H-6ʹʹ, and H-4ʹʹ to H-11ʹʹ suggest that the nitrobenzene group exhibits the *β*-configuration, and the relative configurations of other chiral centers in dibohemamine I are identical to that of dibohemamine C (**10**). Subsequently, the configuration of **2** was confirmed according to its ECD spectrum (Fig. 3).

The molecular formula of dibohemamine K (**3**) is comfirmed as C_29_H_40_O_7_N_4_ via HR-ESIMS, indicating that dibohemamine K contains one additional oxygen atom compared to dibohemamine B (**8**). Its similar NMR data (Table 1) with those of **8** (Table S1) reveals that a methyl group in **8** is replaced by a hydroxymethyl group, proved via the key HMBC from H_2_-4ʹʹʹ to C-5ʹʹʹ/C-3ʹʹʹ/C-2ʹʹʹ. The *E*-configuration of Δ^2ʹʹʹ^ was proved via the NOE correlation between H-2ʹʹʹ and H_2_-4ʹʹʹ (Fig. 2). Furthermore, compounds **3** and **8** share identical absolute configurations at the chiral centers. This was concluded based on the similarity observed in their NOESY and ECD spectra. (Figs. 2 and 3).

**Fig. 4:**
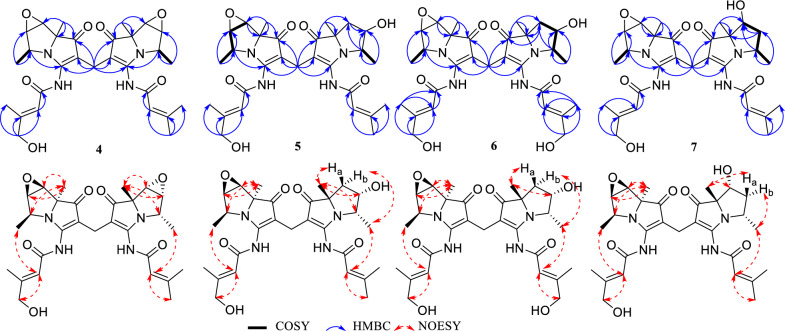
Key 2D NMR correlations of dibohemamines L–O (**4**–**7**)

The molecular formula of **4** was C_29_H_36_O_7_N_4_ (HR-ESIMS *m*/*z* 575.2468, calcd for C_29_H_36_O_7_N_4_Na, 575.24762). The NMR spectra (Figs. S27 − S32) were similar to those of dibohemamine G (**9**), a semisynthetic dibohemamine [13]. The NMR data (Table 2) reveal that a hydroxymethyl group at C-4ʹ or C-4ʹʹʹ in **9** is replaced by a methyl group (*δ*_C/H_ 67.3/4.12). The *E*-configuration of Δ^2′^ was proved via the NOESY of H-2′ to H_2_-4′ (Fig. 4). Therefore, the molecular framework of **4** is established as a heterodimer comprising bohemamine A and bohemamine K, which are linked through a methylene bridge between C-2 and C-2ʹʹ positions. The configuration of the two pyrrolizidine cores within **4** are congruent with those of **9**, based on the similarity of their NOE correlations and ECD curves (Figs. 3 and 4).

**Table 2:** The ^1^H (600 MHz) and ^13^C (150 MHz) NMR data of **4**–**7** in CD_3_OD

No.	**4**		**5**		**6**^**a**^		**7**	
	*δ*_C_, type	*δ*_H_, mult. (*J* in Hz)	*δ*_C_, type	*δ*_H_, mult. (*J* in Hz)	*δ*_C_, type	*δ*_H_, mult. (*J* in Hz)	*δ*_C_, type	*δ*_H_, mult. (*J* in Hz)
1	200.2, C		201.0, C		201.0, C		201.3, C	
2	104.0, C		104.3, C		105.1, C		105.2, C	
3	168.3, C		168.6, C		168.4, C		168.9, C	
4	57.7, CH	3.92, qd (6.6, 3.5)	57.7, CH	3.92, q (6.5)	57.7, CH	3.92, q (6.5)	57.7, CH	3.90, q (6.6)
5	65.4, CH	3.70, dd (3.5, 3.0)	65.4, CH	3.68, d (2.9)	65.4, CH	3.68, d (3.0)	65.3, CH	3.66, d (2.9)
6	56.8, CH	3.63, d (3.0)	56.7, CH	3.62, d (2.9)	56.7, CH	3.62, d (3.0)	56.7, CH	3.60, d (2.9)
7	77.2, C		77.1, C		77.2, C		77.0, C	
8	20.0, CH_3_	1.40, s	19.5, CH_3_	1.42, s	19.7, CH_3_	1.40, s	19.6, CH_3_	1.37, s
9	14.2, CH_3_	1.31, d (6.6)	14.3, CH_3_	1.32, d (6.5)	14.2, CH_3_	1.31, d (6.5)	14.3, CH_3_	1.32, d (6.6)
10	14.4, CH_2_	2.83, s	14.1, CH_2_	2.95, d (15.8) 2.83, d (15.8)	14.1, CH_2_	2.82, d (15.8) 2.91, d (15.8)	14.5, CH_2_	2.87, d (15.6) 2.97, d (15.6)
1ʹ	166.3, C		166.4, C		166.3, C		166.6, C	
2ʹ	115.2, CH	6.33 − 6.34, m	114.9, CH	6.33 − 6.34, m	115.0, CH	6.33 − 6.34, m	115.1, CH	6.33, br s
3ʹ	162.5, C		162.9, C		162.8, C		162.6, C	
4ʹ	67.3, CH_2_	4.12, br s	67.3, CH_2_	4.14, d (1.2)	67.3, CH_2_	4.13, br s	67.3, CH_2_	4.14, br s
5ʹ	16.1, CH_3_	2.14, d (0.7)	16.1, CH_3_	2.16, br s	16.1, CH_3_	2.16, br s	16.1, CH_3_	2.16, br s
1ʹʹ	200.0, C		201.0, C		202.9, C		201.3, C	
2ʹʹ	104.3, C		105.1, C		104.3, C		105.2, C	
3ʹʹ	168.3, C		166.5, C		166.5, C		165.6, C	
4ʹʹ	57.8, CH	3.93, qd (6.6, 3.5)	60.4, CH	4.12, q (6.7)	60.3, CH	4.10, q (6.8)	56.9, CH	4.14, overlap
5ʹʹ	65.4, CH	3.70, dd (3.5, 3.0)	74.2, CH	4.79, ddd (11.8, 6.7, 5.8)	74.2, CH	4.76, ddd (11.8, 6.8, 5.8)	44.8, CH_2_	1.97, overlap 2.87, overlap
6ʹʹ	56.8, CH	3.64, d (3.0)	37.0, CH_2_	1.67, t (11.8) 2.06, dd (11.8, 5.8)	36.9, CH_2_	1.66, t (11.8) 2.02, dd (11.8, 5.8)	74.6, CH	4.19, d (3.6)
7ʹʹ	77.1, C		74.3, C		74.1, C		84.5, C	
8ʹʹ	20.1, CH_3_	1.40, s	25.7, CH_3_	1.40, s	25.8, CH_3_	1.39, s	23.1, CH_3_	1.41, s
9ʹʹ	14.2, CH_3_	1.30, d (6.6)	10.4, CH_3_	0.92, d (6.7)	10.4, CH_3_	0.92, d (6.8)	20.6, CH_3_	1.24, d (6.5)
1ʹʹʹ	165.9, C		164.7, C		162.5, C		164.7, C	
2ʹʹʹ	118.3, CH	6.01 − 6.02, m	118.2, CH	6.09 − 6.10, m	115.1, C	6.40 − 6.41, m	118.2, C	6.07, br s
3ʹʹʹ	160.6, C		161.1, C		165.1, C		161.1, C	
4ʹʹʹ	27.8, CH_3_	1.99, d (1.1)	27.8, CH_3_	2.00, d (1.0)	67.3, CH_2_	4.13, br s	27.7, CH_3_	1.99, br s
5ʹʹʹ	20.7, CH_3_	2.24, d (1.1)	20.7, CH_3_	2.24, d (1.0)	16.1, CH_3_	2.15, br s	20.7, CH_3_	2.23, br s

^a^The chemical shifts of carbons were obtained via DEPTQ experiments

The molecular formula of bohemamine M (**5**) was comfirmed as C_29_H_38_O_7_N_4_ by HR-ESIMS spectrum. This formula indicates an additional oxygen atom in its structure compared to that of dibohemamine F (**11**). The similar NMR data with those of **11** (Tables 3 and S2) reveals that a methyl group in **11** is replaced by a hydroxymethyl group. The HMBC from H_2_-4′ to C-2′/C-3′/C-5′, and the NOESY of H-2′ to H_2_-4′/H_3_-9 suggest a hydroxyl group substitution at the C-4′ position. The absolute configuration of the pyrrolizidine cores of **5** was confirmed to be the same as **11** via the ECD spectra (Fig. 3).

**Table 3:** Cytotoxic activities of compounds **1** and **10**

Compounds	IC_50_ ± SD (*μ*M)	
	786-O	GBC-SD
**1**	3.24 ± 0.20	7.36 ± 0.41
**10**	8.17 ± 1.07	> 10
Dox	0.03 ± 0.01	0.09 ± 0.01

Compound **6** has a molecular formula of C_29_H_38_O_8_N_4_, which has one additional oxygen atom than **5**. The NMR data (Table 2) of **5** and **6** are strikingly similar expect a single methyl signal that is replaced by a hydroxymethyl signal in **6**. The HMBC confirms that C-4′ and C-4′′ positions are substituted by hydroxyl groups. The *E*-configurations of Δ^2′^ and Δ^2′′^ were confirmed via the NOESY of H_2_-4′ to H-2′, and H_2_-4′′′ to H-2′′′, respectively. Furthermore, the absolute configuration of **6** is identical to that of **5**, indicated via similar NOESY correlations (Figs. 4, S40, and S48) and ECD (Fig. 3) spectra.

Compound **7** is also a dibohemamine, with a molecular formula of C_29_H_38_O_7_N_4_. The comparison of its NMR data (Table 2) with the known dibohemamine reveals that it is a hydroxyl substituted dibohemamine D [17]. The hydroxyl substitution at the C-4′ position is suggested via the NOESY correlations of H-2′ to H_2_-4′/H_3_-9, and the HMBC from H-4′ to C-2′/C-3′/C-5′. The absolute configuration of **7** was confirmed to be identical to that of dibohemamine D by the NOESY correlations (Fig. 4) and ECD spectra (Fig. 3).

Compounds **1**–**11** were evaluated for the cytotoxic activity against eighteen cell lines [14, 20]. In which, dibohemamine I (**1**) showed potential cytotoxic activities against human renal clear cell adenocarcinoma (786-O, IC_50_ = 3.24 ± 0.20 μM) and the gallbladder cancer (GBC-SD, IC_50_ = 7.36 ± 0.41 μM) cell lines. Dibohemamine C (**10**) showed specific cytotoxic activities against 786-O with an IC_50_ value of 8.17 ± 1.07 μM.

## Conclusions

Although bohemamine dimers have been successfully semisynthesized through a chemical reaction involving bohemamines and formaldehyde, the production of bohemamines remains dependent on scarce natural sources [15, 16]. The pursuit of bioactive dimers from natural origins remains a paramount strategy for the discovery of novel bioactive dibohemamines. In this study, seven new dibohemamines (**1** − **7**) were obtained from an endophytic *Streptomyces* species in *H. Cordata*. Dibohemamine J (**2**) represents a new skeleton with a nitrobenzene-substituted bohenamine moiety. To date, only six natural and twelve semisynthetic bohemamine dimers have been reported [15–17]. Dibohemamines B–F and four semisynthetic dimers exhibited cytotoxic activity towards non-small cell lung or liver cancer cells. [15, 17] This study reveals that dibohemamines I (**1**) and C (**10**) exhibit potent cytotoxic effects on human renal clear cell adenocarcinoma and gallbladder cancer cell lines, thereby broadening the horizons for the therapeutic applications of these compounds within the realm of oncology.

## Experimental

### General experimental procedures

The equipment employed for acquiring HRESIMS, NMR, UV–Vis, IR, ECD, and ORD data, as well as the HPLC systems utilized for both analysis and separation, are consistent with those detailed in previous studies [14, 20]. UV–Vis, ECD, and ORD were measured in the solutions of MeOH. For semi-preparative HPLC separations, either ODS-A columns (YMC, 5 μm, 1 × 25 cm, 4 mL/min) or πNAP columns (COSMOSIL, 5 μm, 1 × 25 cm, 4 mL/min) were deployed.

### Strain material, fermentation and isolation

Strain GZWMJZ-662 was obtain from the roots of *H. cordata*, [14] and was determined as *Streptomyces* species (16S rRNA, GenBank No. OR083423). The strain was grown on ISP2 liquid cultured medium in 500 mL Erlenmeyer flasks for 3 days (28 °C and 180 rpm) to yield seed liquid. Two hundreds of sterile cultural bags each containing 50 g rice, 10 g soybean powder, 0.25 g sodium chloride and 50 mL water were static culture for one month after seed liquid (9 mL) was added. The culture media were extracted with ethyl acetate (EtOAc) and methanol (MeOH) (10:1, v/v). After evaporated, the residual was redispersed into MeOH (5 L) and washed petroleum ether (PE) (5 L*3 times). Then, the methanol was evaporated to yield duck extract (270 g).

The extract was loaded to a normal-phase silica gel column, and eluted by PE, DCM, and DCM:MeOH (100:1 to 1:1, v/v). After thin-layer chromatography detection and sample combination, 37 fractions (Fr1 − Fr37) were obtained.

Fr.20 (8.9 g) was separated by Sephadex LH-20 (MeOH:DCM = 1:1, v/v)) into Fr.20.1 to Fr.20.10. Fr.20.5 (510 mg) was initially separated by PTLC using EtOAc, yielding Fr.20.5.1 to Fr.20.5.3. Fr.20.5.1 (115 mg) was further separated using HPLC (ODS-A column) to obtain **11** (*t*_R_ = 25.5 min, 8.8 mg) using 60% MeOH-H_2_O containing 0.05% TFA. Fr.20.5.2 (64 mg) was separated by an intelligent flash purification system (C18 column) using a gradient elution of 5% to 100% MeOH-H_2_O (containing 0.1% TFA) to obtain **4** (10.1 mg). Fr.20.5.3 (39 mg) was separated by HPLC using ODS-A column to obtain **1** (*t*_R_ = 66 min, 2.1 mg) using 65% MeOH-H_2_O (containing 0.05% TFA) at 10 mL/min. Fr.20.4 (741 mg) was separated by a flash purification system (C18 column) using a gradient elution of 5% to 100% MeOH-H_2_O (0.1% TFA) to obtain 8 fractions Fr.20.4.1 to Fr.20.4.8). Fr.20.4.8 (27 mg) was separated by HPLC (ODS-A column) to obtain **2** (*t*_R_ = 20.3 min, 2.2 mg) using 60% MeOH-H_2_O (0.05% TFA).

Fr.25 (7.5 g) was separated by Sephadex LH-20 into 9 fractions (Fr.25.1 − Fr.25.9) using MeOH and DCM (1:1, v/v). Fr.25.7 (564 mg) was separated by a C18 flash chromatography column using a gradient elution of 5% to 100% MeOH-H_2_O (0.1% TFA) to obtain 8 subfractions (Fr.25.7.1 − Fr.25.7.8). Fr.25.7.1 (41 mg) was washed with MeOH and DCM separately, resulting in a white insoluble substance **9** (7.8 mg). Fr.25.7.6 (49 mg) was separated by a semi-preparative πNAP column to obtain **10** (*t*_R_ = 18.0 min, 5.5 mg) using 80% MeOH-H_2_O (containing 0.05% TFA). Fr.25.7.4 (44 mg) was separated by a semi-preparative πNAP column to obtain **7** (*t*_R_ = 25.5 min, 3.5 mg) using 65% MeOH-H_2_O (0.05% TFA).

Fr.28 (4.714 g) was separated by Sephadex LH-20 into 9 fractions (Fr.28.1 − Fr.28.9) using MeOH and DCM (1:1, v/v) as the eluent. Fr.28.6 (226.7 mg) was further separated by Toyopearl HW-40F resin into 2 fractions (Fr.28.6.1 and Fr.28.6.2) using methanol as the eluent. Fr.28.6.1 (93 mg) was separated by an ODS-A chromatography column to obtain **8** (*t*_R_ = 14.3 min, 8.7 mg) using 75% MeOH-H_2_O (containing 0.05% TFA). Similarly, Fr.28.6.2 (103 mg) was separated by a semi-preparative ODS-A chromatography column to obtain **5** (*t*_R_ = 18 min, 20.9 mg) using 55% MeOH-H_2_O (0.05% TFA).

Fr.30 (8.791 g) was separated by Sephadex LH-20 into 9 fractions (Fr.30.1 − Fr.30.9) using equal volume of MeOH and DCM as eluent. Fr.30.6 (1.9 g) was further subjected to a C18 flash chromatography column (5% to 100% MeOH-H_2_O, containing 0.1% TFA) to obtain 15 fractions (Fr.30.6.1 − Fr.30.6.15). Fr.30.6.4 (98 mg) was then purified by semi-preparative HPLC (ODS-A) to obtain **6** (*t*_R_ = 12.0 min, 5.1 mg) using 50% MeOH-H_2_O (containing 0.05% TFA).

Fr.31 (2.7 g) was separated by Toyopearl HW-40F gel resin into 11 fractions (Fr.31.1 − Fr.31.11) using equal volume of MeOH and DCM the eluent. Fr.31.4 (408 mg) underwent initial separation by a Flash chromatography column (C18) using a gradient elution of 5% to 100% MeOH-H_2_O (containing 0.1% TFA) to obtain 4 fractions (Fr.31.4.1 − Fr.31.4.4). Fr.31.4.1 (120 mg) was further purified by a semi-preparative ODS-A chromatography column to obtain **3** (*t*_R_ = 12.4 min, 28.4 mg) using 65% MeOH-H_2_O (containing 0.05% TFA).

### Spectroscopic data of compounds

#### Dibohemamine I (1)

White powder; $[\alpha]_\text{D}^{25}$ =  − 79.0 (*c* 0.1); UV–Vis *λ*_max_(log*ε*) 248 (4.45), 285 (4.18), 346 (4.11) nm; IR (KBr) *ν*_max_: 3272, 3151, 2979, 2930, 1712, 1692, 1646, 1554, 1451, 1217, 1132, 1073, 1013, 925, 845, 800, 724, 664, 582 cm^−1^; ECD (0.87 mM) *λ*_max_ (Δε) 246 (+ 22.6), 285 (− 61.1), 345 (+ 15.9) nm; chemical shifts of ^1^H and ^13^C (Table 1); HR-ESIMS *m*/*z* 597.2433 [M + Na]^+^ (calcd for C_29_H_39_O_6_N_4_ClNa 597.24503).

#### Dibohemamine J (2)

White powder; $[\alpha]_\text{D}^{25}$ =  − 167.0 (*c* 0.1); UV–Vis *λ*_max_ (log *ε*) 247 (4.16), 288 (3.84), 344 (3.81) nm; IR (KBr) *ν*_max_: 3273, 2979, 2933, 1682, 1646, 1567, 1495, 1446, 1383, 1206, 1183, 1133, 1064, 1011, 842, 801, 722, 664, 587, 544 cm^−1^; ECD (0.76 mM) *λ*_max_ (Δε) 248 (+ 15.0), 288 (− 54.8), 345 (+ 15.5) nm; chemical shifts of ^1^H and ^13^C (Table 1); HR-ESIMS *m*/*z* 662.3174 [M + H]^+^ (calcd for C_35_H_44_O_8_N_5_, 662.31844).

#### Dibohemamine K (3)

White powder; $[\alpha]_\text{D}^{25}$ =  − 78.0 (*c* 0.1); UV–Vis *λ*_max_ (log *ε*) *λ*_max_(log*ε*) 249 (4.50), 284 (4.23), 346 (4.20) nm; IR (KBr) *ν*_max_: 3275, 3217, 2980, 2934, 1712, 1692, 1643, 1554, 1449, 1375, 1331, 1214, 1183, 1135, 1074, 1017, 926, 898, 843, 801, 760, 724, 661, 587 cm^−1^; ECD (0.22 mM) *λ*_max_ (Δε) 248 (+ 28.7), 285 (− 71.2), 343 (+ 18.7) nm; chemical shifts of ^1^H and ^13^C (Table 1); HR-ESIMS *m*/*z* 557.2946 [M + H]^+^ (calcd for C_29_H_41_O_7_N_4_, 557.29698).

#### Dibohemamine L (4)

White powder; $[\alpha]_\text{D}^{25}$ =  − 110.0 (*c* 0.1); UV–Vis *λ*_max_(log*ε*) 246 (4.51), 285 (4.22), 347 (4.19) nm; IR (KBr) *ν*_max_: 3210, 3031, 2977, 2916, 1708, 1646, 1567, 1492, 1455, 1370, 1324, 1214, 1123, 1053, 1015, 902, 843, 700.1, 674, 587, 530 cm^−1^; ECD (0.45 mM) *λ*_max_ (Δε) 245 (+ 34.0), 285 (− 92.8), 347 (+ 30.2) nm; chemical shifts of ^1^H and ^13^C (Table 2); HR-ESIMS *m*/*z* 575.2468 [M + Na]^+^ (calcd for C_29_H_36_O_7_N_4_Na, 575.24762).

#### Dibohemamine M (5)

White powder; $[\alpha]_\text{D}^{25}$ =  − 87.0 (*c* 0.1); UV–Vis *λ*_max_(log*ε*) 247 (4.47), 285 (4.20), 346 (4.19) nm; IR (KBr) *ν*_max_: 3273, 3148, 2980, 2936, 1708, 1683, 1647, 1554, 1452, 1372, 1324, 1211, 1185, 1135, 1074, 1015, 929, 906, 843, 801, 724, 662, 590 cm^−1^; ECD (0.23 mM) *λ*_max_ (Δε) 245 (+ 21.6), 286 (− 56.4), 346 (+ 16.5) nm; chemical shifts of ^1^H and ^13^C (Table 2); HR-ESIMS *m*/*z* 555.2809 [M + H]^+^ (calcd for C_29_H_39_O_7_N_4_,555.28133).

#### Dibohemamine N (6)

White powder; $[\alpha]_\text{D}^{25}$ =  − 113.3 (*c* 0.1); UV–Vis *λ*_max_(log*ε*) 249 (4.50), 284 (4.23), 346 (4.20) nm; IR (KBr) *ν*_max_: 3273, 3210, 2982, 2933, 2834, 1712, 1693, 1674, 1550, 1492, 1455, 1399, 1372, 1326, 1214, 1176, 1123, 1079, 1054, 1017, 977, 928, 902, 845, 774, 699, 668, 586 cm^−1^; ECD (0.22 mM) *λ*_max_ (Δε) 244 (+ 32.9), 285 (− 68.6), 346 (+ 28.4) nm; chemical shifts of ^1^H and ^13^C (Table 2); HR-ESIMS *m*/*z* 571.2740 [M + H]^+^ (calcd for C_29_H_39_O_8_N_4_, 571.27624).

#### Dibohemamine O (7)

White powder; $[\alpha]_\text{D}^{25}$ =  − 175.3 (*c* 0.1); UV–Vis *λ*_max_(log*ε*) 248 (4.46), 285 (4.17), 347 (4.13) nm; IR (KBr) *ν*_max_: 3273, 3210, 2982, 2939, 1683, 1647, 1545, 1512, 1446, 1377, 1326, 1208, 1135, 1067, 1011, 929, 905, 843, 801, 724, 668, 579, 536 cm^−1^; ECD (0.90 mM) *λ*_max_ (Δε) 244 (+ 27.4), 284 (− 66.4), 348 (+ 29.2) nm; chemical shifts of ^1^H and ^13^C (Table 2); HR-ESIMS *m*/*z* 555.2791 [M + H]^+^ (calcd for C_29_H_39_O_7_N_4_, 555.28133).

### Cytotoxicity assay

The cytotoxic activity of **1**–**11** against eighteen cell lines were assayed as our described previously [14, 20]. The tested cell lines see Table S3.

## Supplementary Information

Supplementary Material 1.

## Data Availability

The datasets used or analysed during the current study are available from the corresponding author on reasonable request.
