# Supplementary material for: Dibohemamines I–O from Streptomyces sp. GZWMJZ-662, an endophytic actinomycete from the medicinal and edible plant Houttuynia cordata Thunb
Source: Nat Prod Bioprospect. 2025 Jan 6;15(1):9. doi: 10.1007/s13659-024-00494-4 (PMC11703785; doi:10.1007/s13659-024-00494-4)
Supplement: Supplementary file 1 — Supplementary Material 1. [file 13659_2024_494_MOESM1_ESM.docx]

**Dibohemamines I–O from *Streptomyces* sp. GZWMJZ-662, an Endophytic Actinomycete from the Medicinal and Edible Plant *Houttuynia cordata* Thunb.**

Dong-Yang Wang^1,2†^, Ming-Xing Li^1,2†^, Yan-Chao Xu^1,3^, Peng Fu^4^, Wei-Ming Zhu^1,4*^, and Li-Ping Wang^1,2*^

**Affiliation**

^1^ State Key Laboratory of Functions and Applications of Medicinal Plants, Guizhou Medical University, Guiyang 550014, China.

^2^ Natural Product Research Center of Guizhou Province, Guiyang 550014, China.

^3^ School of Pharmaceutical Sciences, Guizhou Medical University, Guiyang 561113, China.

^4^ School of Medicine and Pharmacy, Ocean University of China, Qingdao, 266003, China.

^†^ Contributed equally to this work

^*^Corresponding authors:

E-mail: [weimingzhu@ouc.edu.cn](mailto:weimingzhu@ouc.edu.cn) (W.M. Zhu); [wangliping2022@gmc.edu.cn](mailto:wangliping2022@gmc.edu.cn) (L.P. Wang)

List of Supporting Information

[**The physical properties of 8-11** S4](#_Toc182491663)

[**Table S1.** The ^1^H (600 MHz) and ^13^C (150 MHz) NMR data of compounds **8** and **9** S4](#_Toc182491664)

[**Table S2.** The ^1^H (600 MHz) and ^13^C (150 MHz) NMR data of **10** and **11** S5](#_Toc182491665)

[**Table S3.** The tested cell lines S6](#_Toc182491666)

[**Figure S1.** IR spectrum of compound **1** S7](#_Toc182491667)

[**Figure S2.** HRESIMS spectrum of compound **1** S8](#_Toc182491668)

[**Figure S3.** ^1^H NMR spectrum of compound **1** in CD_3_OD (600 MHz) S9](#_Toc182491669)

[**Figure S4.** DEPTQ spectrum of compound **1** in CD_3_OD (150 MHz) S10](#_Toc182491670)

[**Figure S5.** ^1^H-^1^H COSY spectrum of compound **1** in CD_3_OD (600 MHz) S11](#_Toc182491671)

[**Figure S6.** HSQC spectrum of compound **1** in CD_3_OD (600x150 MHz) S12](#_Toc182491672)

[**Figure S7.** HMBC spectrum of compound **1** in CD_3_OD (600x150 MHz) S13](#_Toc182491673)

[**Figure S8.** NOESY spectrum of compound **1** in CD_3_OD (600 MHz) S14](#_Toc182491674)

[**Figure S9.** IR spectrum of compound **2** S15](#_Toc182491675)

[**Figure S10.** HRESIMS spectrum of compound **2** S16](#_Toc182491676)

[**Figure S11.** ^1^H NMR spectrum of compound **2** in CD_3_OD (600 MHz) S17](#_Toc182491677)

[**Figure S12.** DEPTQ spectrum of compound **2** in CD_3_OD (150 MHz) S18](#_Toc182491678)

[**Figure S13.** ^1^H-^1^H COSY spectrum of compound **2** in CD_3_OD (600 MHz) S19](#_Toc182491679)

[**Figure S14.** HSQC spectrum of compound **2** in CD_3_OD (600x150 MHz) S20](#_Toc182491680)

[**Figure S15.** HMBC spectrum of compound **2** in CD_3_OD (600x150 MHz) S21](#_Toc182491681)

[**Figure S16.** NOESY spectrum of compound **2** in CD_3_OD (600 MHz) S22](#_Toc182491682)

[**Figure S17.** IR spectrum of compound **3** S23](#_Toc182491683)

[**Figure S18.** HRESIMS spectrum of compound **3** S24](#_Toc182491684)

[**Figure S19.** ^1^H NMR spectrum of compound **3** in CD_3_OD (600 MHz) S25](#_Toc182491685)

[**Figure S20.** DEPTQ spectrum of compound **3** in CD_3_OD (150 MHz) S26](#_Toc182491686)

[**Figure S21.** ^1^H-^1^H COSY spectrum of compound **3** in CD_3_OD (600 MHz) S27](#_Toc182491687)

[**Figure S22.** HSQC spectrum of compound **3** in CD_3_OD (600x150 MHz) S28](#_Toc182491688)

[**Figure S23.** HMBC spectrum of compound **3** in CD_3_OD (600x150 MHz) S29](#_Toc182491689)

[**Figure S24.** NOESY spectrum of compound **3** in CD_3_OD (600 MHz) S30](#_Toc182491690)

[**Figure S25.** IR spectrum of compound **4** S31](#_Toc182491691)

[**Figure S26.** HRESIMS spectrum of compound **4** S32](#_Toc182491692)

[**Figure S27.** ^1^H NMR spectrum of compound **4** in CD_3_OD (600 MHz) S33](#_Toc182491693)

[**Figure S28.** ^13^C NMR spectrum of compound **4** in CD_3_OD (150 MHz) S34](#_Toc182491694)

[**Figure S29.** ^1^H-^1^H COSY spectrum of compound **4** in CD_3_OD (600 MHz) S35](#_Toc182491695)

[**Figure S30.** HSQC spectrum of compound **4** in CD_3_OD (600x150 MHz) S36](#_Toc182491696)

[**Figure S31.** HMBC spectrum of compound **4** in CD_3_OD (600x150 MHz) S37](#_Toc182491697)

[**Figure S32.** NOESY spectrum of compound **4** in CD_3_OD (600 MHz) S38](#_Toc182491698)

[**Figure S33.** IR spectrum of compound **5** S39](#_Toc182491699)

[**Figure S34.** HRESIMS spectrum of compound **5** S40](#_Toc182491700)

[**Figure S35.** ^1^H NMR spectrum of compound **5** in CD_3_OD (600 MHz) S41](#_Toc182491701)

[**Figure S36.** ^13^C NMR spectrum of compound **5** in CD_3_OD (150 MHz) S42](#_Toc182491702)

[**Figure S37.** ^1^H-^1^H COSY spectrum of compound **5** in CD_3_OD (600 MHz) S43](#_Toc182491703)

[**Figure S38.** HSQC spectrum of compound **5** in CD_3_OD (600x150 MHz) S44](#_Toc182491704)

[**Figure S39.** HMBC spectrum of compound **5** in CD_3_OD (600x150 MHz) S45](#_Toc182491705)

[**Figure S40.** NOESY spectrum of compound **5** in CD_3_OD (600 MHz) S46](#_Toc182491706)

[**Figure S41.** IR spectrum of compound **6** S47](#_Toc182491707)

[**Figure S42.** HRESIMS spectrum of compound **6** S48](#_Toc182491708)

[**Figure S43.** ^1^H NMR spectrum of compound **6** in CD_3_OD (600 MHz) S49](#_Toc182491709)

[**Figure S44.** DEPTQ spectrum of compound **6** in CD_3_OD (150 MHz) S50](#_Toc182491710)

[**Figure S45.** ^1^H-^1^H COSY spectrum of compound **6** in CD_3_OD (600 MHz) S51](#_Toc182491711)

[**Figure S46.** HSQC spectrum of compound **6** in CD_3_OD (600x150 MHz) S52](#_Toc182491712)

[**Figure S47.** HMBC spectrum of compound **6** in CD_3_OD (600x150 MHz) S53](#_Toc182491713)

[**Figure S48.** NOESY spectrum of compound **6** in CD_3_OD (600 MHz) S54](#_Toc182491714)

[**Figure S49.** IR spectrum of compound **7** S55](#_Toc182491715)

[**Figure S50.** HRESIMS spectrum of compound **7** S56](#_Toc182491716)

[**Figure S51.** ^1^H NMR spectrum of compound **7** in CD_3_OD (600 MHz) S57](#_Toc182491717)

[**Figure S52.** ^13^C NMR spectrum of compound **7** in CD_3_OD (150 MHz) S58](#_Toc182491718)

[**Figure S53.** ^1^H-^1^H COSY spectrum of compound **7** in CD_3_OD (600 MHz) S59](#_Toc182491719)

[**Figure S54.** HSQC spectrum of compound **7** in CD_3_OD (600x150 MHz) S60](#_Toc182491720)

[**Figure S55.** HMBC spectrum of compound **7** in CD_3_OD (600x150 MHz) S61](#_Toc182491721)

[**Figure S56.** NOESY spectrum of compound **7** in CD_3_OD (600 MHz) S62](#_Toc182491722)

**The physical properties of 8-11**

*Dibohemamine B (****8****):* white powder; [α]25 D = −84.0 (*c* 0.1, MeOH); UV (MeOH) *λ*_max_(log*ε*) 249 (4.39), 284 (4.08), 345 (3.14) nm; ECD (0.23 mM, MeOH) *λ*_max_ (Δε) 246 (+21.9), 285 (−53.4), 344 (+14.3) nm; ^1^H and ^13^C NMR see Table S1; IR (KBr) *ν*_max_: 3275, 3210, 3122, 2980, 2934, 1713, 1693, 1642, 1555, 1449, 1369, 1222, 1183, 1135, 1074, 1017, 926, 896, 845, 801, 757, 722, 659, 586 cm^−1^; ESIMS *m*/*z* 541.3 [M + H]^+^.

*Dibohemamine G (****9****):* white powder; [α]25 D = −63.3 (*c* 0.1, MeOH); UV (MeOH) *λ*_max_(log*ε*) 249 (4.59), 289 (4.17), 346 (4.30) nm; ECD (0.22 mM, MeOH) *λ*_max_ (Δε) 245 (+24.6), 296 (−76.8), 351 (+33.8) nm; ^1^H and ^13^C NMR see Table S1; IR (KBr) *ν*_max_: 3210, 2977, 2920, 2832, 1709, 1646, 1622, 1550, 1495, 1454, 1399, 1359, 1326, 1214, 1120, 1053, 1017, 903, 865, 842, 773, 702, 674, 587, 534 cm^−1^; ESIMS *m*/*z* 569.3 [M + H]^+^.

*Dibohemamine C (****10****):* white powder; [α]25 D = −55.3 (*c* 0.1, MeOH); UV (MeOH) *λ*_max_(log*ε*) 249 (4.40), 280 (4.08), 345 (4.09) nm; ECD (0.93 mM, MeOH) *λ*_max_ (Δε) 244 (+34.9), 284 (−64.4), 348 (+23.6) nm; ^1^H and ^13^C NMR see Table S2; IR (KBr) *ν*_max_: 3273, 3124, 2982, 2943, 1683, 1642, 1550, 1449, 1380, 1209, 1136, 1067, 1015, 843, 803, 724, 665, 580 cm^−1^; ESIMS *m*/*z* 541.3 [M + H]^+^.

*Dibohemamine F (****11****):* white powder; [α]25 D = −110.0 (*c* 0.1, MeOH); UV (MeOH) *λ*_max_(log*ε*) 248 (4.58), 283 (4.24), 346 (4.28) nm; ECD (0.23 mM, MeOH) *λ*_max_ (Δε) 245(+29.8), 285 (−76.4), 347 (+21.2) nm; ^1^H and ^13^C NMR see Table S2; IR (KBr) *ν*_max_: 3273, 3214, 2982, 2936, 1708, 1683, 1642, 1554, 1449, 1377, 1323, 1212, 1185, 1136, 1069, 1014, 929, 906, 843, 803, 724, 662, 590 cm^−1^; ESIMS *m*/*z* 539.2 [M + H]^+^.

**Table S1.** The ^1^H (600 MHz) and ^13^C (150 MHz) NMR data of compounds **8** and **9**

|  | **8** in CDCl_3_ | | **8** in CD_3_OD | | **9** in DMSO-*d*_6_ | |
| --- | --- | --- | --- | --- | --- | --- |
| No. | *δ*_C_, type | *δ*_H_, mult. (*J* in Hz) | *δ*_C_, type | *δ*_H_, mult. (*J* in Hz) | *δ*_C_, type | *δ*_H_, mult. (*J* in Hz) |
| 1/1'' | 203.9, C |  | 204.2, C |  | 198.5, C |  |
| 2/2'' | 104.4, C |  | 105.0, C |  | 102.6, C |  |
| 3/3'' | 165.4, C |  | 166.8, C |  | 165.4, C |  |
| 4/4'' | 58.9, CH | 4.11, qd (6.6, 6.0) | 60.3, CH | 4.07, qd (6.7, 6.0) | 55.5, CH | 3.77, q (6.5) |
| 5/5'' | 73.9, CH | 4.72, dt (10.3, 6.0) | 74.3, CH | 4.71, ddd (11.6, 6.0, 5.8) | 65.4, CH | 3.70, dd (2.9) |
| 6/6'' | 36.7, CH_2_ | 2.05, dd (12.1, 6.0)  1.66, dd (12.1, 10.3) | 37.1, CH_2_ | 2.01, dd (11.6, 5.8)  1.62, t (11.6) | 64.0, CH | 3.62, d (2.9) |
| 7/7'' | 73.0, C |  | 74.2, C |  | 74.5, C |  |
| 8/8'' | 25.8, CH_3_ | 1.41, s | 25.8, CH_3_ | 1.39, s | 19.4, CH_3_ | 1.20, s |
| 9/9'' | 9.7, CH_3_ | 0.91, d (6.6) | 10.3, CH_3_ | 0.88, d (6.7) | 15.5, CH_3_ | 1.17, d (6.5) |
| 1'/1''' | 163.7, C |  | 164.9, C |  | 163.9, C |  |
| 2'/2''' | 117.5, CH | 6.09, br s | 118.1, CH | 6.12-6.13, m | 114.2, CH | 6.23, br s |
| 3'/3''' | 159.5, C |  | 161.2, C |  | 159.3, C |  |
| 4'/4''' | 20.8, CH_3_ | 2.25, br s | 20.7, CH_3_ | 2.27 d (0.9) | 55.4, CH_2_ | 3.96, s |
| 5'/5''' | 28.0, CH_3_ | 1.99, br s | 27.8, CH_3_ | 2.03 d (0.9) | 15.5, CH_3_ | 2.03, br s |
| 10 | 13.2, CH_2_ | 2.79, s | 13.8, CH_2_ | 2.88, s | 13.7, CH_2_ | 2.61, s |
| 3/3''-NH |  | 10.10, s |  | 9.71, s |  | 10.00, s |
| 4'/4'''-OH |  |  |  |  |  | 5.29, br s |

**Table S2.** The ^1^H (600 MHz) and ^13^C (150 MHz) NMR data of **10** and **11**

| No. | **10** in CDCl_3_ | | **10** in CD_3_OD | | **11** in CDCl_3_ | | **11** in CD_3_OD | |
| --- | --- | --- | --- | --- | --- | --- | --- | --- |
|  | *δ*_C_, type | *δ*_H_, mult. (*J* in Hz) | *δ*_C_, type | *δ*_H_, mult. (*J* in Hz) | *δ*_C_, type | *δ*_H_, mult. (*J* in Hz) | *δ*_C_, type | *δ*_H_, mult. (*J* in Hz) |
| 1 | 203.5, C |  | 204.7, C |  | 199.8, C |  | 201.0, C |  |
| 2 | 103.2, C |  | 105.3, C |  | 104.5, C |  | 104.5, C |  |
| 3 | 165.7, C |  | 165.8, C |  | 165.5, C |  | 166.6, C |  |
| 4 | 59.3, CH | 4.14, qd (6.6, 6.1) | 60.4, CH | 4.02, qd (6.7, 6.0) | 56.3, CH | 3.97, q (6.5) | 56.7, CH | 3.92, q (6.5) |
| 5 | 73.9, CH | 4.72, ddd (10.3, 6.1, 5.6) | 74.1, CH | 4.67, ddd (10.5, 6.0, 5.7) | 64.4, CH | 3.64, d (2.9) | 65.4, CH | 3.69, d (2.9) |
| 6 | 36.6, CH_2_ | 1.69, dd (11.9, 10.3)  2.07, dd (11.9, 5.6) | 37.1, CH_2_ | 1.62, dd (12.0, 10.5)  1.98, dd (12.0, 5.7) | 56.1, CH | 3.59, d (2.9) | 60.4, CH | 3.64, d (2.9) |
| 7 | 74.3, C |  | 74.2, C |  | 76.3, C |  | 77.4, C |  |
| 8 | 25.7, CH_3_ | 1.49, s | 25.8, CH_3_ | 1.39, s | 19.8, CH_3_ | 1.50, s | 19.7, CH_3_ | 1.41, s |
| 9 | 9.6, CH_3_ | 0.90, d (6.6) | 10.5, CH_3_ | 0.90, d (6.7) | 14.1, CH_3_ | 1.32, d (6.5) | 14.1, CH_3_ | 1.29, d (6.5) |
| 10 | 12.8, CH_2_ | 2.84, d (15.8)  2.95, d (15.8) | 13.9, CH_2_ | 2.90, d (15.7)  2.94, d (15.7) | 13.0, CH_2_ | 2.76, d (15.6)  2.80, d (15.6) | 13.9, CH_2_ | 2.79, d (15.7)  2.88, d (15.7) |
| 1ʹ | 163.3, C |  | 164.8, C |  | 164.6, C |  | 166.0, C |  |
| 2ʹ | 116.9, CH | 5.91, s | 118.0, CH | 6.10-6.11, m | 117.5, CH | 6.07, s | 118.0, CH | 6.05, s |
| 3ʹ | 161.7, C |  | 161.4, C |  | 159.4, C |  | 161.2, C |  |
| 4ʹ | 21.0, CH_3_ | 2.24, s | 27.7, CH_3_ | 2.03, d (1.4) | 28.0, CH_3_ | 2.01, s | 27.8, CH_3_ | 2.26, s |
| 5ʹ | 28.1, CH_3_ | 1.96, s | 20.6, CH_3_ | 2.27, d (1.1) | 20.7, CH_3_ | 2.28, s | 20.6, CH_3_ | 2.02, s |
| 1ʹʹ | 197.9, C |  | 199.6, C |  | 204.2, C |  | 202.7, C |  |
| 2ʹʹ | 107.1, C |  | 106.1, C |  | 104.6, C |  | 105.1, C |  |
| 3ʹʹ | 165.5, C |  | 167.2, C |  | 166.5, C |  | 168.4, C |  |
| 4ʹʹ | 56.1, CH | 4.21, dq (9.2, 6.5) | 56.7, CH | 4.12, dq (9.2, 6.5) | 59.1, CH | 4.16, qd (6.7, 6.1) | 57.7, CH | 4.10, qd (6.7, 6.3) |
| 5ʹʹ | 43.2, CH_2_ | 1.99, d (13.7)  2.80, ddd (13.7, 9.2, 4.3) | 44.9, CH_2_ | 1.95, d (14.1)  2.85, ddd (14.1, 9.2, 6.8) | 73.0, CH | 4.75, dt (11.3, 6.1) | 74.2, CH | 4.76, ddd (12.6, 11.3, 6.3) |
| 6ʹʹ | 73.5, CH | 4.20, d (4.3) | 74.6, CH | 4.16, d (6.8) | 36.7, CH_2_ | 1.76, dd (11.6, 11.3)  2.09, dd (11.6, 6.1) | 36.9, CH_2_ | 1.64, dd (11.5, 11.3)  2.03, dd (12.6, 11.5) |
| 7ʹʹ | 84.7, C |  | 84.4, C |  | 73.0, C |  | 74.1, C |  |
| 8ʹʹ | 22.9, CH_3_ | 1.43, s | 22.9, CH_3_ | 1.36, s | 25.9, CH_3_ | 1.44, s | 25.8, CH_3_ | 1.39, s |
| 9ʹʹ | 20.1, CH_3_ | 1.19, d (6.5) | 20.7, CH_3_ | 1.21, d (6.5) | 9.7, CH_3_ | 1.00, d (6.7) | 10.3, CH_3_ | 0.89, d (6.7) |
| 1ʹʹʹ | 163.2, C |  | 165.2, C |  | 163.7, C |  | 164.7, C |  |
| 2ʹʹʹ | 116.9, CH | 6.14, s | 118.3, CH | 6.12-6.13, m | 117.6, CH | 6.13, s | 118.2, CH | 6.11, s |
| 3ʹʹʹ | 160.6, C |  | 160.8, C |  | 159.2, C |  | 161.0, C |  |
| 4ʹʹʹ | 20.7, CH_3_ | 2.27, s | 27.8, CH_3_ | 2.04, d (1.4) | 28.0, CH_3_ | 2.03, s | 27.8, CH_3_ | 2.26, s |
| 5ʹʹʹ | 27.8, CH_3_ | 2.03, s | 20.6, CH_3_ | 2.26, d (1.1) | 20.7, CH_3_ | 2.29, s | 20.6, CH_3_ | 2.02, s |
| 3-NH |  | 10.66, s |  |  |  | 9.96, s |  |  |
| 3ʹʹ-NH |  | 9.44, s |  |  |  | 10.00, s |  |  |

**Table S3.** The tested cell lines

| Cell number | Cell name | Cell number | Cell name |
| --- | --- | --- | --- |
| MKN-45 | Human gastric cancer cell line | SF126 | Human brain tumor cell line |
| HCT116 | Human colon cancer cell line | DU145 | Human prostate cancer cell line |
| HeLa | Human cervical cancer cell line | CAL-62 | Human thyroid cancer cell line |
| K562 | Human chronic myeloid leukemia cell line | PATU8988T | Human pancreatic cancer cell line |
| 786-O | Human renal clear cell adenocarcinoma cell line | HOS | Human osteosarcoma cell line |
| TE-1 | Human esophageal cancer cell line | A-375 | Human malignant melanoma cell line |
| 5637 | Human bladder cancer cell line | A-673 | Human rhabdomyosarcoma cell line |
| GBC-SD | Human gallbladder cancer cell line | L-02 | Human normal liver cell line |
| MCF-7 | Human breast cancer cell line | 293T | Human kidney cell line |

**Figure S1.** IR spectrum of compound **1**


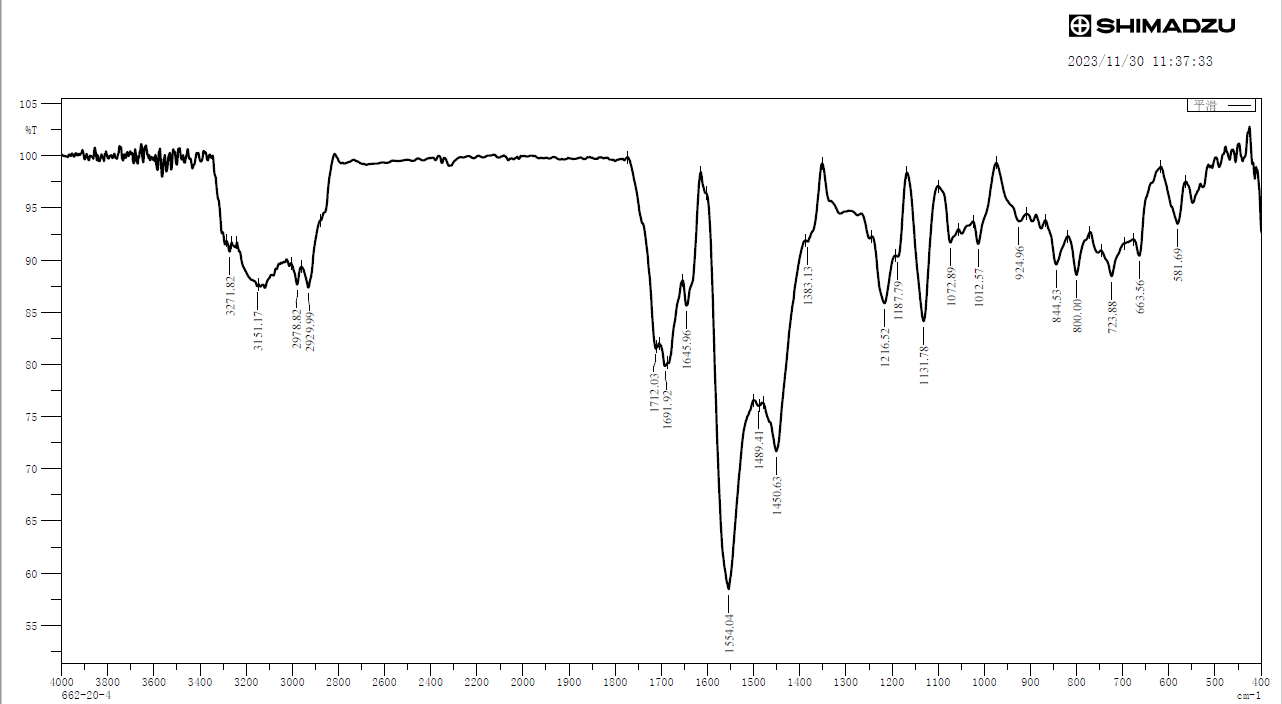

**Figure S2.** HRESIMS spectrum of compound **1**


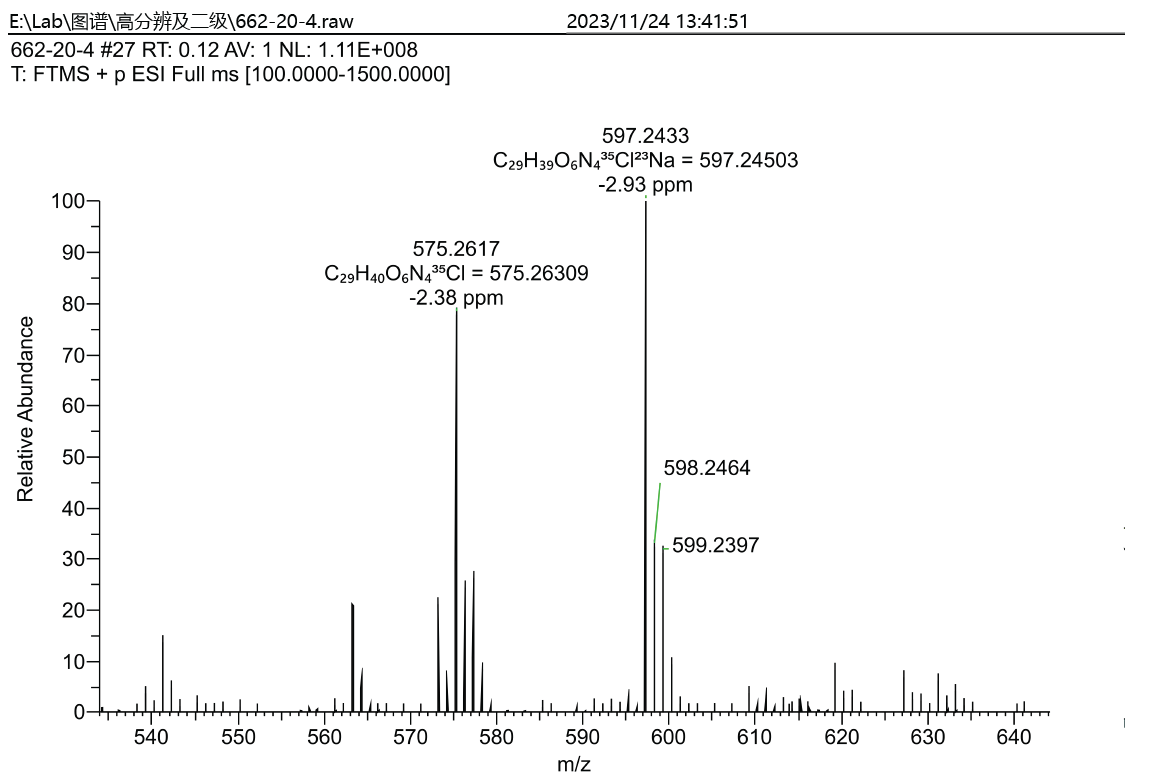

**Figure S3.** ^1^H NMR spectrum of compound **1** in CD_3_OD (600 MHz)

**Figure S4.** DEPTQ spectrum of compound **1** in CD_3_OD (150 MHz)

**Figure S5.** ^1^H-^1^H COSY spectrum of compound **1** in CD_3_OD (600 MHz)

**Figure S6.** HSQC spectrum of compound **1** in CD_3_OD (600x150 MHz)

**Figure S7.** HMBC spectrum of compound **1** in CD_3_OD (600x150 MHz)

**Figure S8.** NOESY spectrum of compound **1** in CD_3_OD (600 MHz)

**Figure S9.** IR spectrum of compound **2**


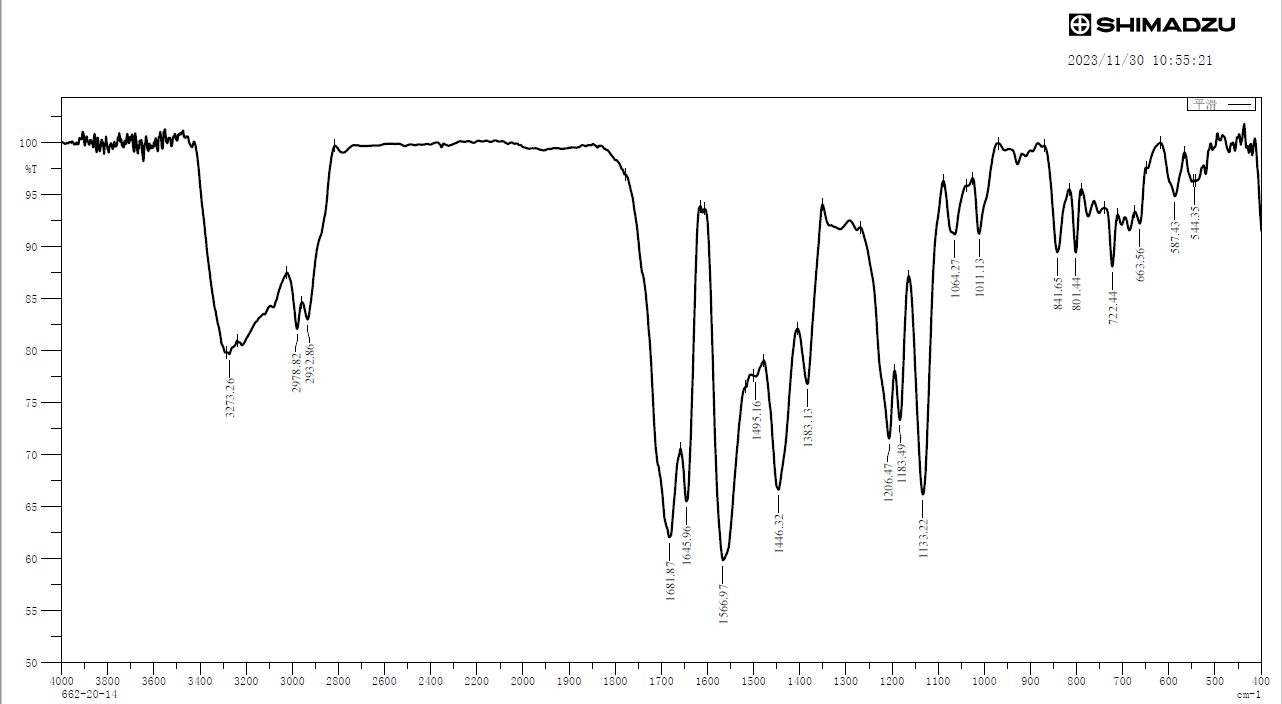

**Figure S10.** HRESIMS spectrum of compound **2**


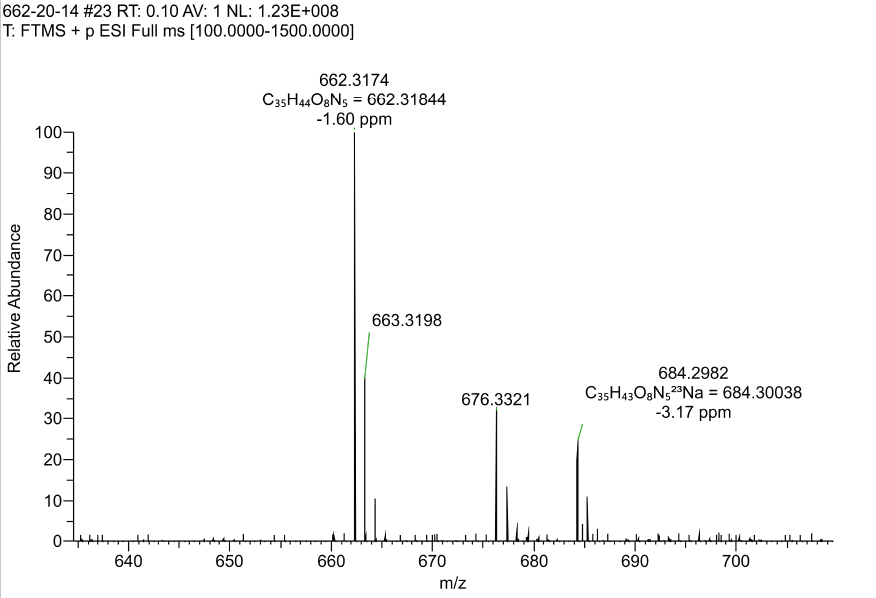

**Figure S11.** ^1^H NMR spectrum of compound **2** in CD_3_OD (600 MHz)

**Figure S12.** DEPTQ spectrum of compound **2** in CD_3_OD (150 MHz)

**Figure S13.** ^1^H-^1^H COSY spectrum of compound **2** in CD_3_OD (600 MHz)

**Figure S14.** HSQC spectrum of compound **2** in CD_3_OD (600x150 MHz)

**Figure S15.** HMBC spectrum of compound **2** in CD_3_OD (600x150 MHz)

**Figure S16.** NOESY spectrum of compound **2** in CD_3_OD (600 MHz)

**Figure S17.** IR spectrum of compound **3**


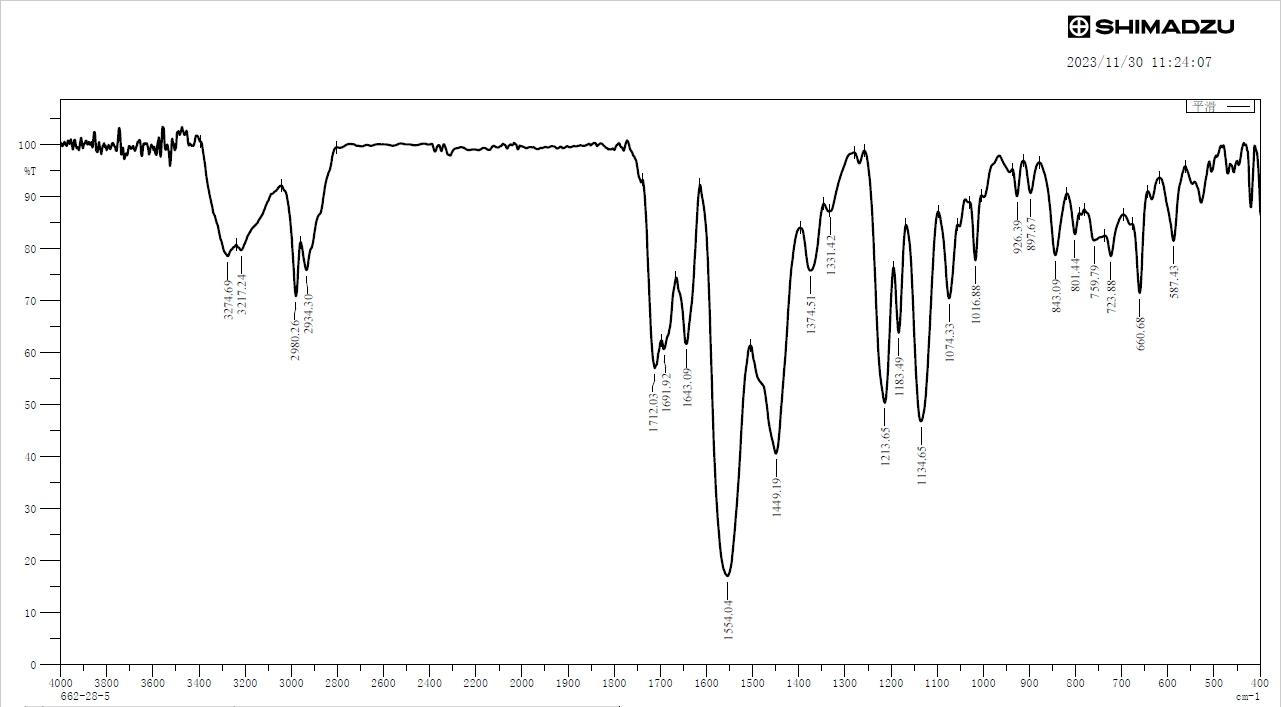

**Figure S18.** HRESIMS spectrum of compound **3**


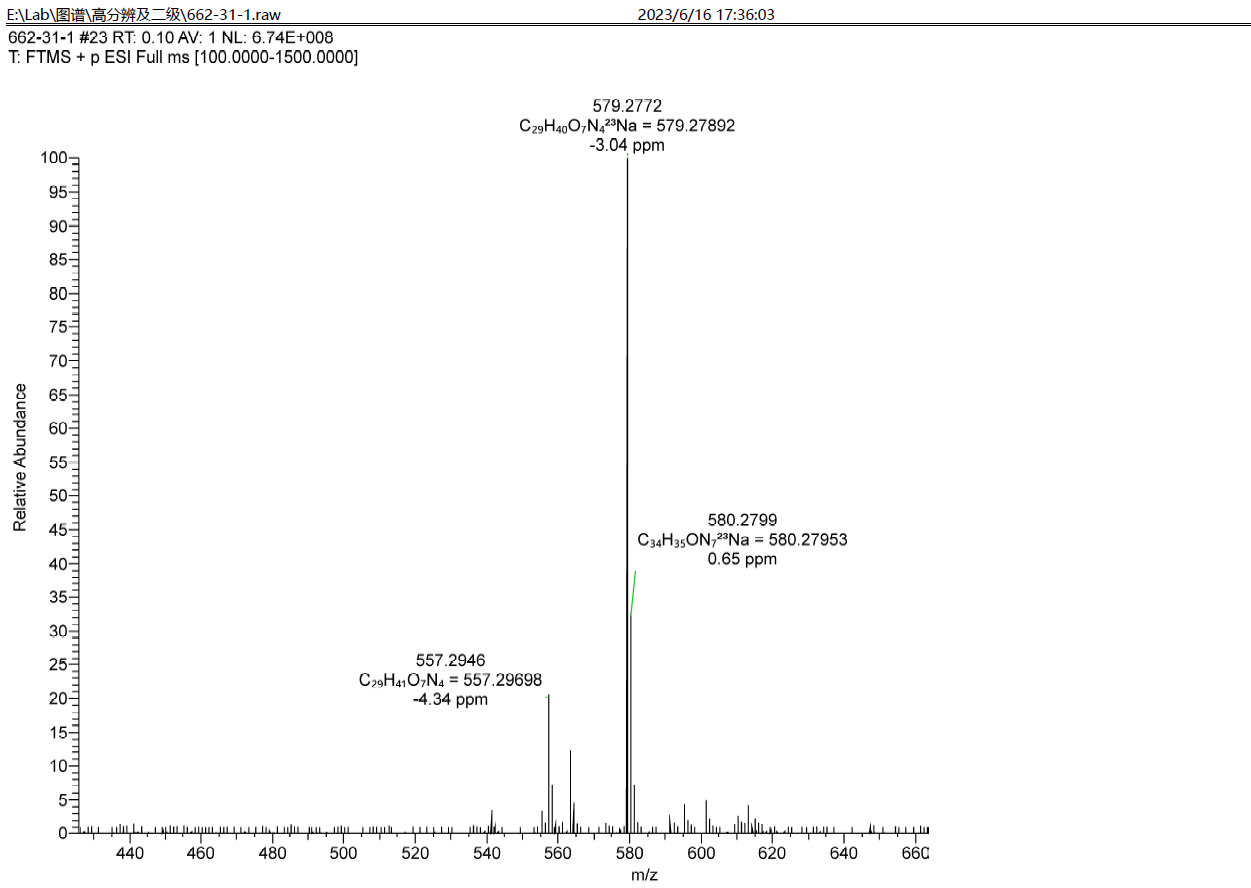

**Figure S19.** ^1^H NMR spectrum of compound **3** in CD_3_OD (600 MHz)

**Figure S20.** DEPTQ spectrum of compound **3** in CD_3_OD (150 MHz)

**Figure S21.** ^1^H-^1^H COSY spectrum of compound **3** in CD_3_OD (600 MHz)

**Figure S22.** HSQC spectrum of compound **3** in CD_3_OD (600x150 MHz)

**Figure S23.** HMBC spectrum of compound **3** in CD_3_OD (600x150 MHz)

**Figure S24.** NOESY spectrum of compound **3** in CD_3_OD (600 MHz)

**Figure S25.** IR spectrum of compound **4**


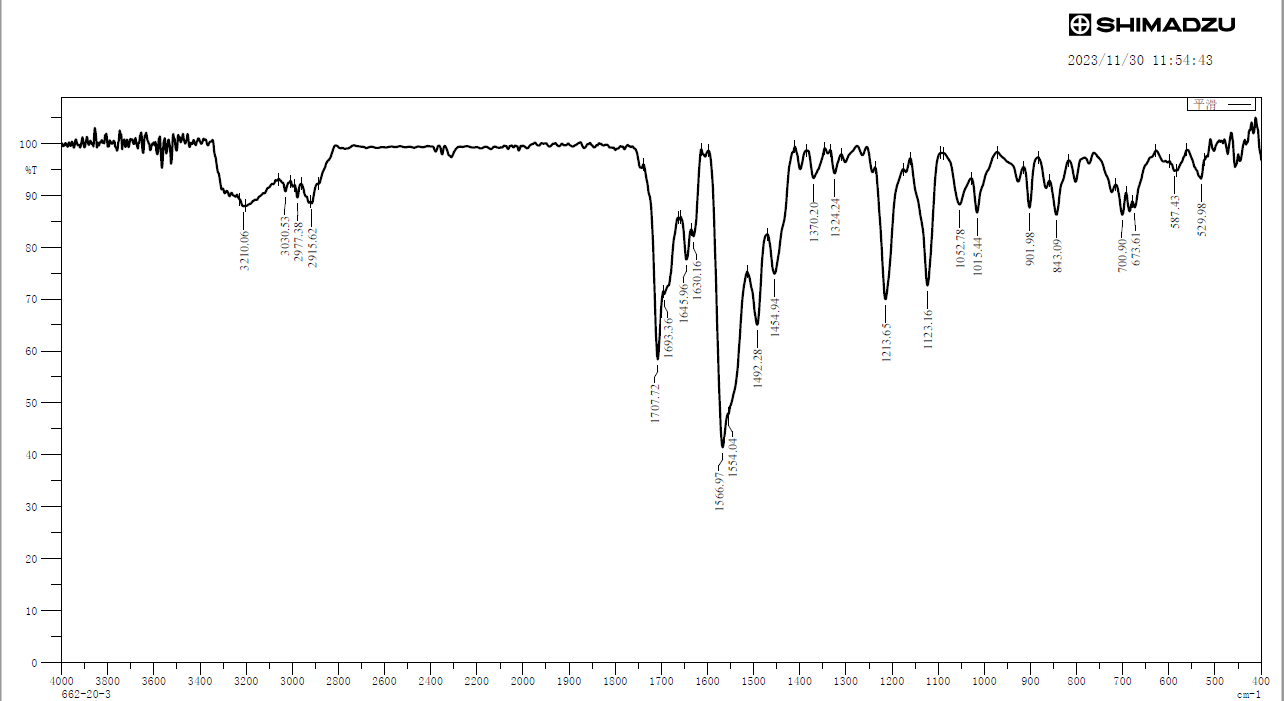

**Figure S26.** HRESIMS spectrum of compound **4**


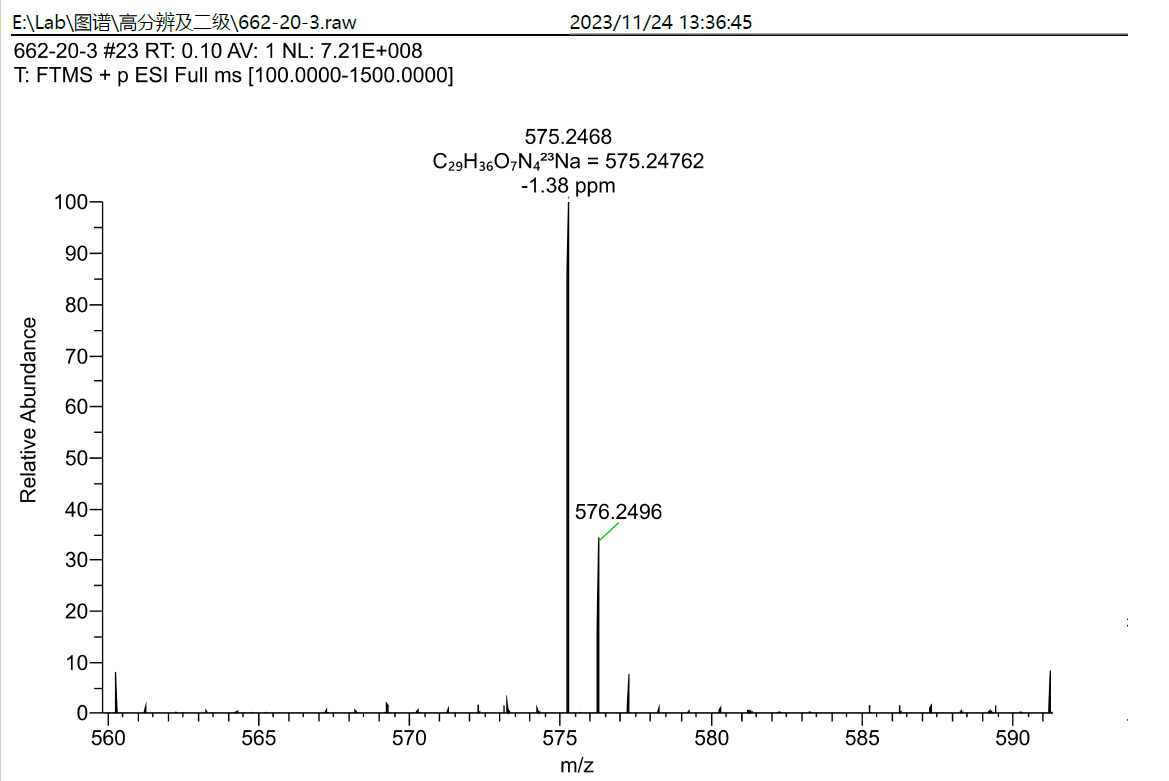

**Figure S27.** ^1^H NMR spectrum of compound **4** in CD_3_OD (600 MHz)

**Figure S28.** ^13^C NMR spectrum of compound **4** in CD_3_OD (150 MHz)

**Figure S29.** ^1^H-^1^H COSY spectrum of compound **4** in CD_3_OD (600 MHz)

**Figure S30.** HSQC spectrum of compound **4** in CD_3_OD (600x150 MHz)

**Figure S31.** HMBC spectrum of compound **4** in CD_3_OD (600x150 MHz)

**Figure S32.** NOESY spectrum of compound **4** in CD_3_OD (600 MHz)

**Figure S33.** IR spectrum of compound **5**


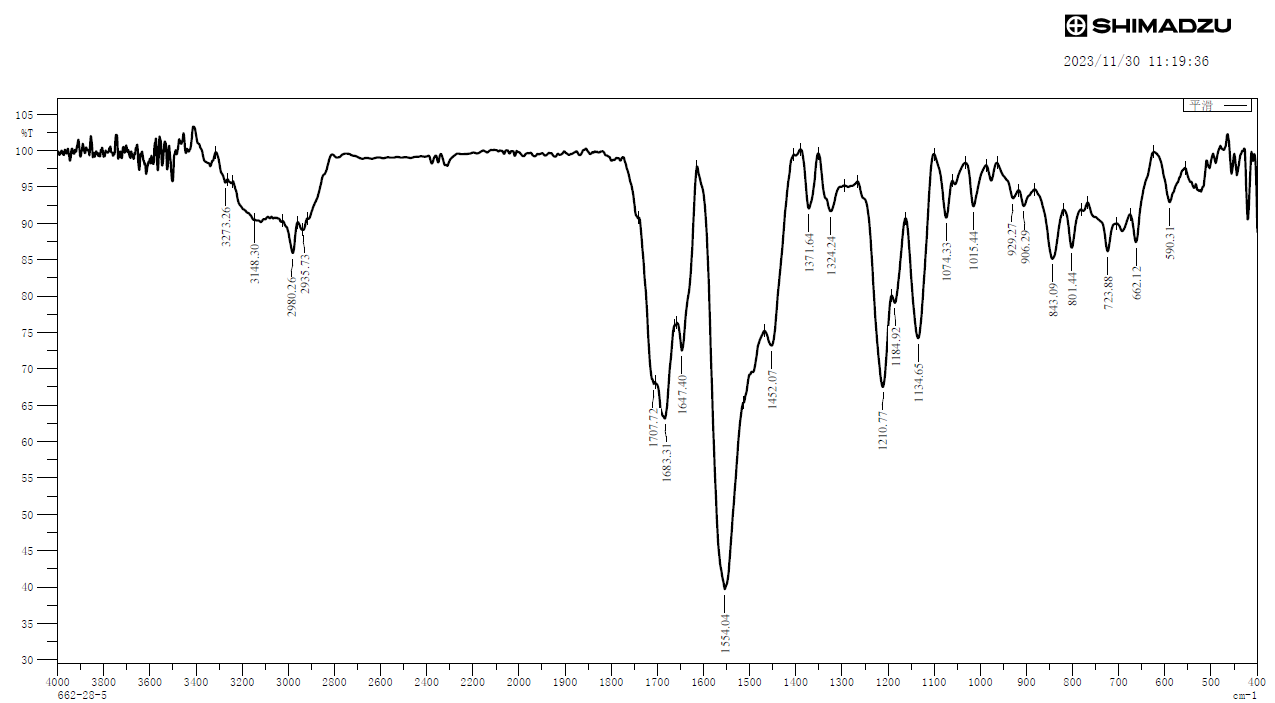

**Figure S34.** HRESIMS spectrum of compound **5**


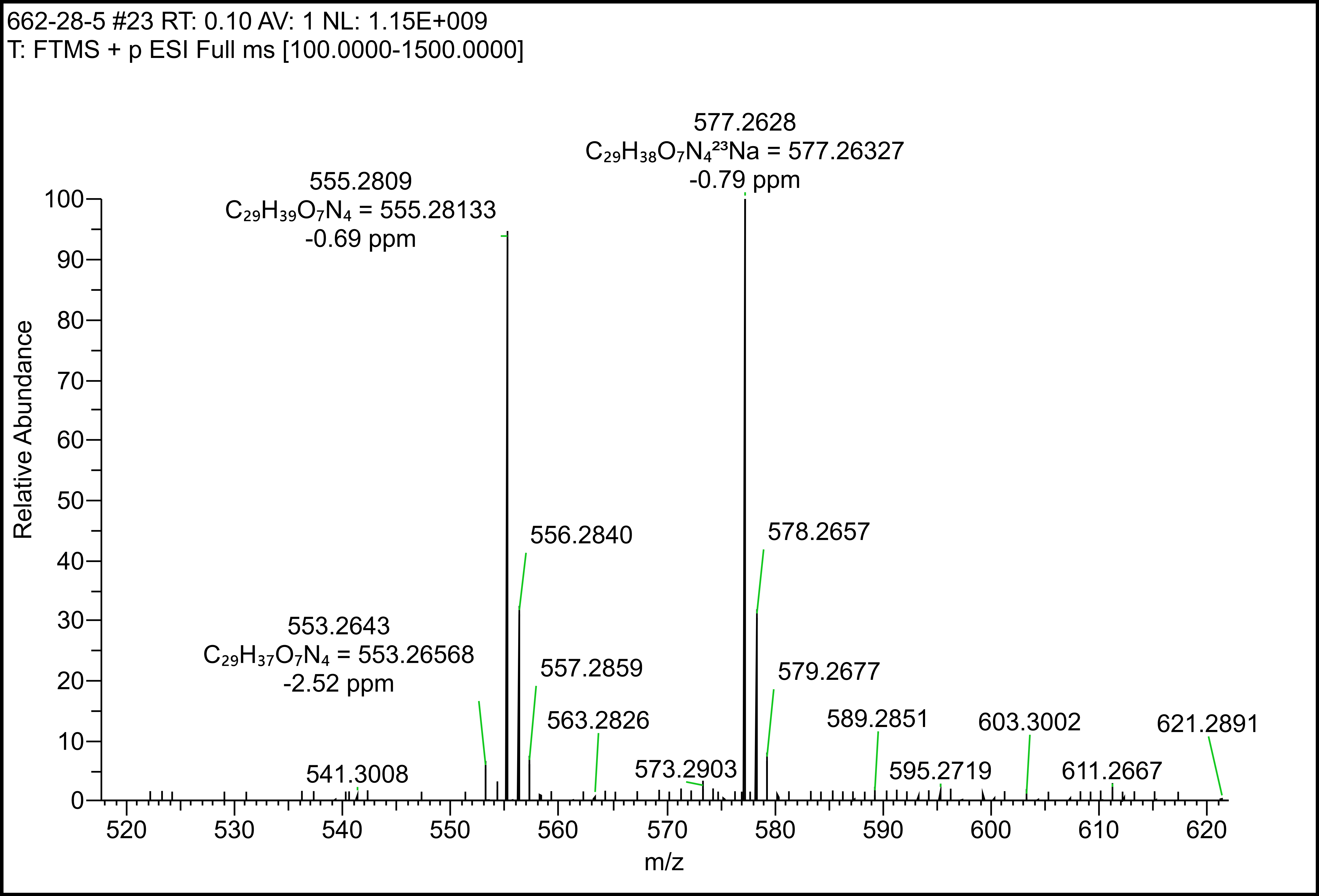

**Figure S35.** ^1^H NMR spectrum of compound **5** in CD_3_OD (600 MHz)

**Figure S36.** ^13^C NMR spectrum of compound **5** in CD_3_OD (150 MHz)

**Figure S37.** ^1^H-^1^H COSY spectrum of compound **5** in CD_3_OD (600 MHz)

**Figure S38.** HSQC spectrum of compound **5** in CD_3_OD (600x150 MHz)

**Figure S39.** HMBC spectrum of compound **5** in CD_3_OD (600x150 MHz)

**Figure S40.** NOESY spectrum of compound **5** in CD_3_OD (600 MHz)

**Figure S41.** IR spectrum of compound **6**


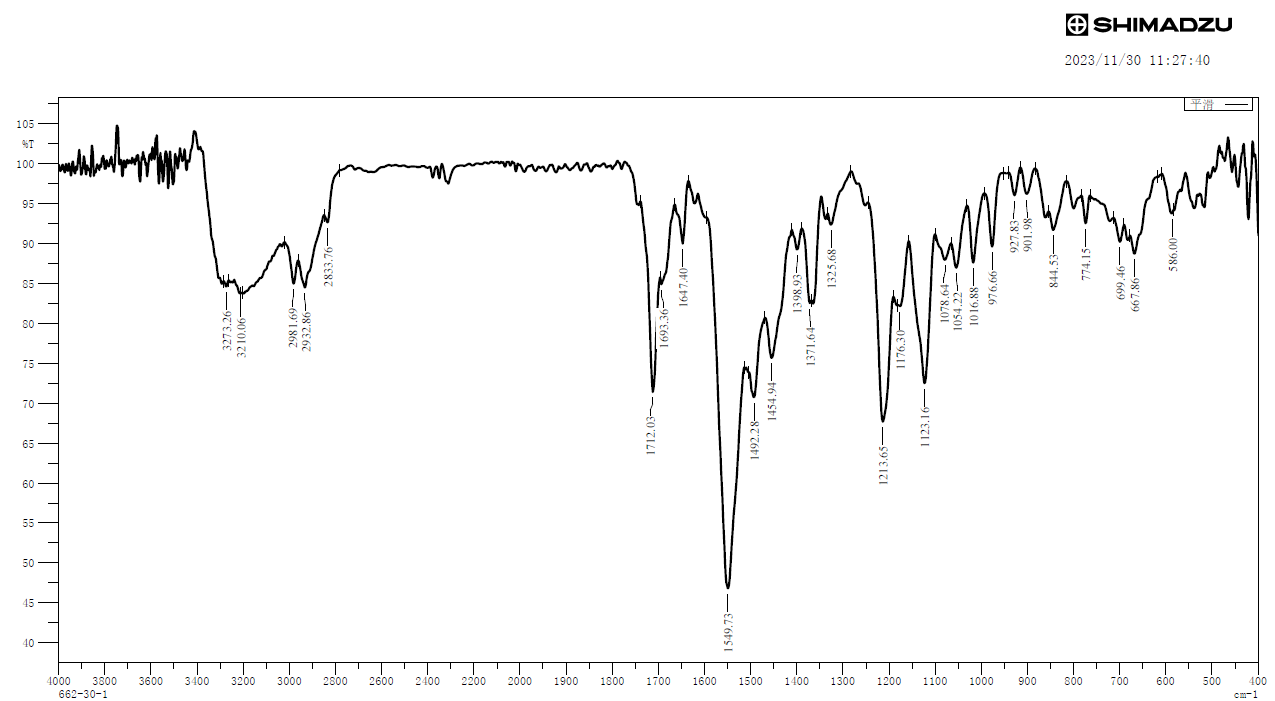

**Figure S42.** HRESIMS spectrum of compound **6**


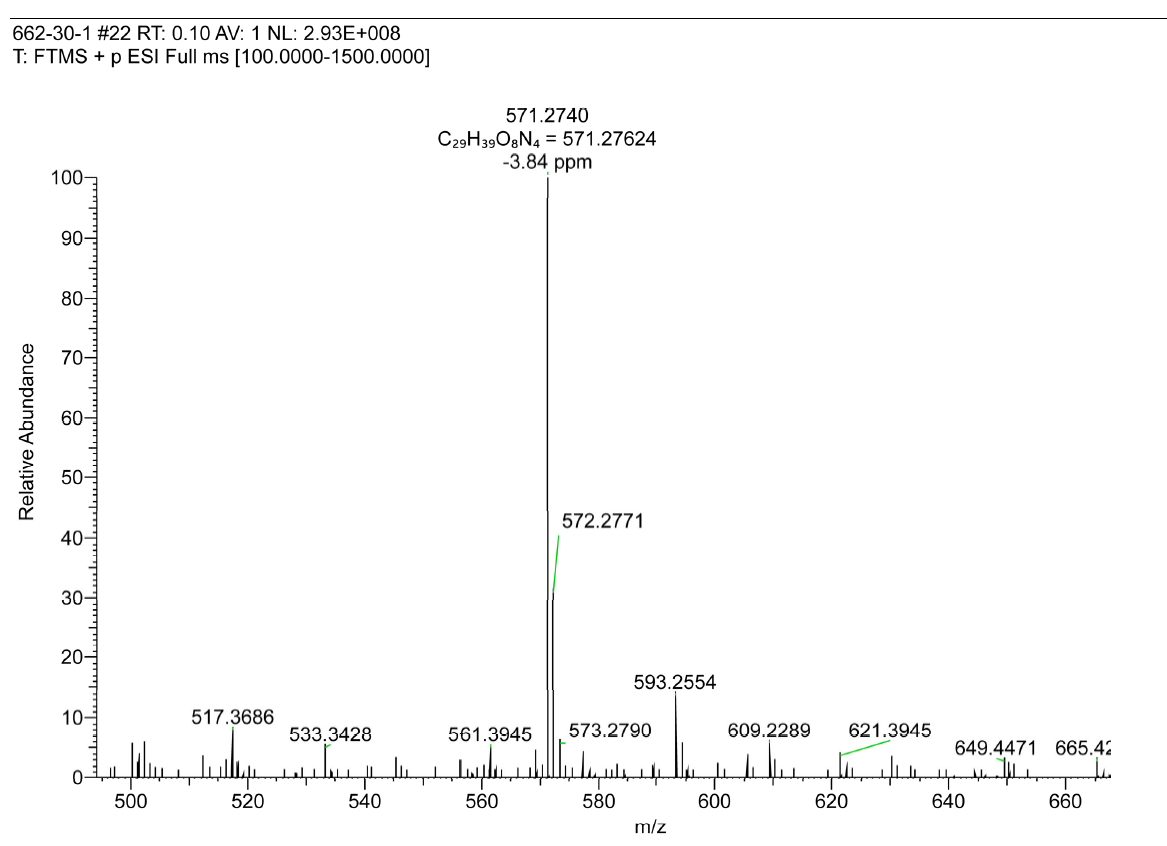

**Figure S43.** ^1^H NMR spectrum of compound **6** in CD_3_OD (600 MHz)

**Figure S44.** DEPTQ spectrum of compound **6** in CD_3_OD (150 MHz)

**Figure S45.** ^1^H-^1^H COSY spectrum of compound **6** in CD_3_OD (600 MHz)

**Figure S46.** HSQC spectrum of compound **6** in CD_3_OD (600x150 MHz)

**Figure S47.** HMBC spectrum of compound **6** in CD_3_OD (600x150 MHz)

**Figure S48.** NOESY spectrum of compound **6** in CD_3_OD (600 MHz)

**Figure S49.** IR spectrum of compound **7**


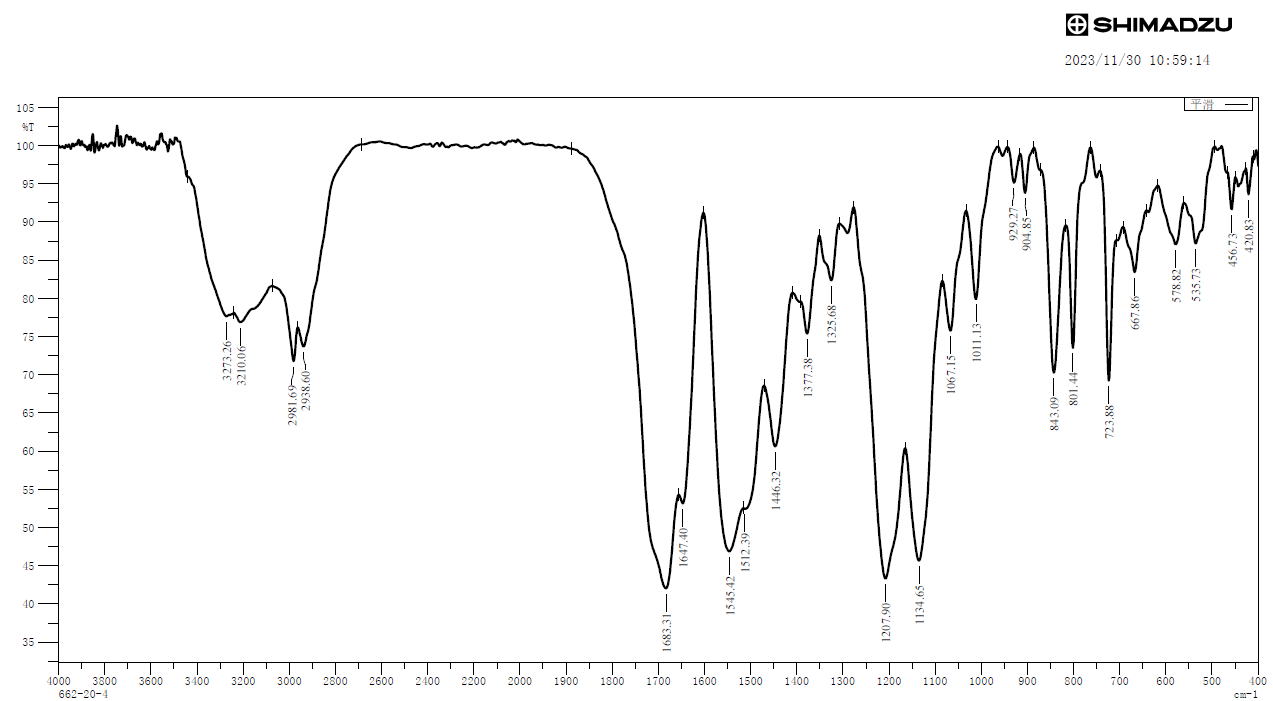

**Figure S50.** HRESIMS spectrum of compound **7**


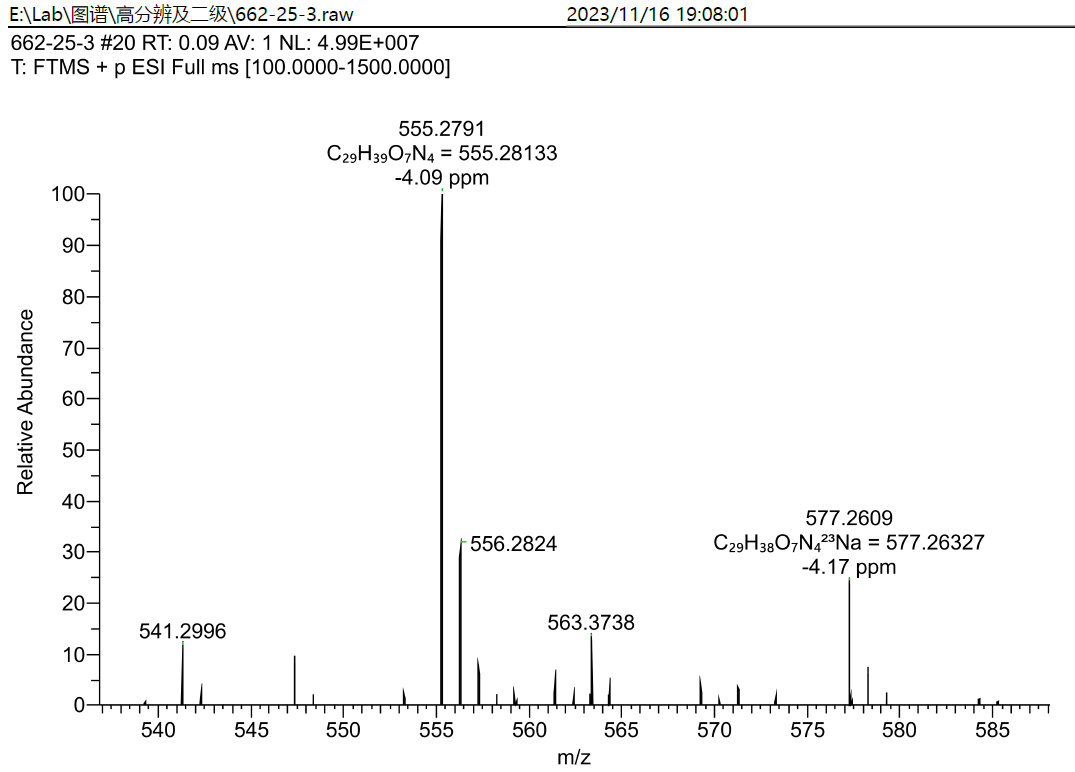

**Figure S51.** ^1^H NMR spectrum of compound **7** in CD_3_OD (600 MHz)

**Figure S52.** ^13^C NMR spectrum of compound **7** in CD_3_OD (150 MHz)

**Figure S53.** ^1^H-^1^H COSY spectrum of compound **7** in CD_3_OD (600 MHz)

**Figure S54.** HSQC spectrum of compound **7** in CD_3_OD (600x150 MHz)

**Figure S55.** HMBC spectrum of compound **7** in CD_3_OD (600x150 MHz)

**Figure S56.** NOESY spectrum of compound **7** in CD_3_OD (600 MHz)
